# Survey of Datafusion Techniques for Laser and Vision Based Sensor Integration for Autonomous Navigation

**DOI:** 10.3390/s20082180

**Published:** 2020-04-12

**Authors:** Prasanna Kolar, Patrick Benavidez, Mo Jamshidi

**Affiliations:** Department of Electrical Engineering, the University of Texas at San Antonio, 1, UTSA Cir., San Antonio, TX 78249, USA

**Keywords:** datafusion, data fusion, multimodal, fusion, information fusion, survey, review, RGB, SLAM, localization, obstacle detection, obstacle avoidance, navigation, deep learning, neural networks, mapping, LiDAR, optical, vision, stereo vision, autonomous systems, data integration, data alignment, robot, mobile robot

## Abstract

This paper focuses on data fusion, which is fundamental to one of the most important modules in any autonomous system: perception. Over the past decade, there has been a surge in the usage of smart/autonomous mobility systems. Such systems can be used in various areas of life like safe mobility for the disabled, senior citizens, and so on and are dependent on accurate sensor information in order to function optimally. This information may be from a single sensor or a suite of sensors with the same or different modalities. We review various types of sensors, their data, and the need for fusion of the data with each other to output the best data for the task at hand, which in this case is autonomous navigation. In order to obtain such accurate data, we need to have optimal technology to read the sensor data, process the data, eliminate or at least reduce the noise and then use the data for the required tasks. We present a survey of the current data processing techniques that implement data fusion using different sensors like LiDAR that use light scan technology, stereo/depth cameras, Red Green Blue monocular (RGB) and Time-of-flight (TOF) cameras that use optical technology and review the efficiency of using fused data from multiple sensors rather than a single sensor in autonomous navigation tasks like mapping, obstacle detection, and avoidance or localization. This survey will provide sensor information to researchers who intend to accomplish the task of motion control of a robot and detail the use of LiDAR and cameras to accomplish robot navigation.

## 1. Introduction

Autonomous systems can play a vital role in assisting humans in a variety of problem areas. This could potentially be in a wide range of applications like driver-less cars, humanoid robots, assistive systems, domestic systems, military systems, and manipulator systems, to name a few. Presently, the world is at a bleeding edge of technologies that can enable this even in our daily lives. Assistive robotics is a crucial area of autonomous systems that helps persons who require medical, mobility, domestic, physical, and mental assistance. This research area is gaining popularity in applications like autonomous wheelchair systems [1,2], autonomous walkers [3], lawn movers [4,5], vacuum cleaners [6], intelligent canes [7], and surveillance systems in places like assisted living [8,9,10,11].

Data are one of the most important components to optimally start, continue, or complete any task. Often, these data are obtained from the environment that the autonomous system functions in; examples of such data could be the system’s position and location coordinates in the environment, the static objects, speed/velocity/acceleration of the system or its peers or any moving object in its vicinity, vehicle heading, air pressure, and so on. Since this is obtained directly from the operational environment, the information is up-to-date and can be accessed through either built-in or connected sensing equipment/devices. This survey is focused on the vehicle navigation of an autonomous vehicle. We review the past and present research using Light Imaging Detection and Ranging (LiDAR) and Imaging systems like a camera, which are laser and vision-based sensors, respectively. The autonomous systems use sensor data for tasks like object detection, obstacle avoidance, mapping, localization, etc. As we will see in the upcoming sections, these two sensors can complement each other and hence are being used extensively for detection in autonomous systems. The LiDAR market alone is expected to reach USD $52.5 Billion by the year 2032, as given in a recent survey by the Yole group, documented by “First Sensors” group [12].

**Figure 1:**
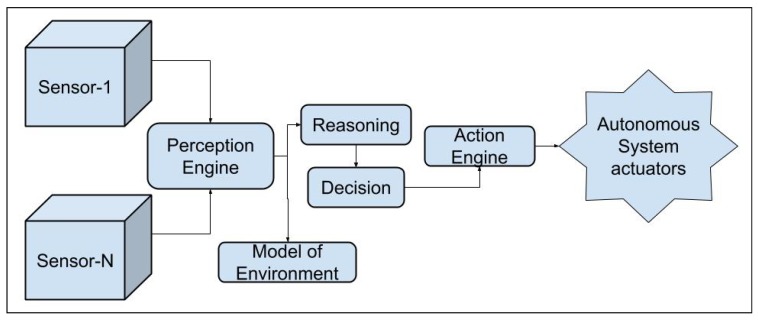
High level Perception Architecture.

In a typical autonomous system, a perception module inputs the optimal information into the control module. Refer Figure 1. Crowley et al. [13] define perception.

The process of maintaining an internal description of the external environment.

This paper is organized as follows:

This section, Section 1 introduces autonomous systems and how data fusion is used. Section 2 introduces data fusion, techniques, need and compares single vs. multi sensor fusion. Section 3 discusses some of hardware that could be used for autonomous navigation. Section 4 reviews autonomous vehicle navigation. It considers mapping, localization, and obstacle avoidance. Section 5 details how data fusion is used in autonomous navigation. Section 6 gives the conclusions after reviewing the present research.

## 2. Data Fusion

Data fusion entails combining information to accomplish something. This ’something’ is usually to sense the state of some aspect of the universe [14]. The applications of this ’state sensing’ are versatile, to say the least. Some high level areas are: neurology, biology, sociology, engineering, physics, and so on [15,16,17,18,19,20,21]. Due to the very versatile nature of the application of data fusion, throughout this manuscript, we will limit our review to the usage of data fusion using LiDAR data and camera data for autonomous navigation. More information about data fusion will be provided in the upcoming sections.

### 2.1. Sensors and Their Input to Perception

A sensor is an electronic device that measures physical aspects of an environment and outputs machine (a digital computer) readable data. They provide a direct perception of the environment they are implemented in. Typically, a suite of sensors is used since it is the inherent property of an individual sensor, in order to provide a single aspect of an environment. This not only enables the completeness of the data, but also improves the accuracy of measuring the environment.

The Merriam-Webster dictionary defines a sensor [22] as

*A device that responds to a physical stimulus (such as heat, light, sound, pressure, magnetism, or a particular motion) and transmits a resulting impulse (as for measurement or operating a control)*.

The Collins dictionary defines a sensor as [23]:

*A sensor is an instrument which reacts to certain physical conditions or impressions such as heat or light, and which is used to provide information*.

Many applications require multiple sensors to be present to achieve a task. This gives rise to the technique of data fusion, wherein the user will need to provide guidelines and rules for the best usage of the data that is given by the sensors. Several researchers have given their definition of data fusion. JDL’s definition of data fusion is quoted by Hall et al. [24] as:

*A process dealing with the association, correlation, and combination of data and information from single and multiple sources to achieve refined position and identity estimates, and complete and timely assessments of situations and threats, and their significance. The process is characterized by continuous refinements of its estimates and assessments, and the evaluation of the need for additional sources, or modification of the process itself, to achieve improved results*.

Stating that the JDL definition is too restrictive, Hall et al. [21,25,26] re-define data fusion as:

Data fusion is the process of combining data or information to estimate or predict entity states.

*Data fusion involves combining data—in the broadest sense—to estimate or predict the state of some aspect of the universe*.

In addition to the sensors like LiDAR and Camera that are the focus in this survey, any sensor like sonar, stereo vision, monocular vision, radar, LiDAR, etc. can be used in data fusion. Data fusion at this high level will enable tracking moving objects as well, as given in the research conducted by Garcia et al. [27].

The initial step is raw data capture using the sensors. The data is then filtered and an appropriate fusion technology implemented this is fed into localization and mapping techniques like SLAM; the same data can be used to identify static or moving objects in the environment and this data can be used to classify the objects, wherein classification information is used to finalize information in creating a model of the environment which in turn can be fed into the control algorithm [27]. The classification information could potentially give details of pedestrians, furniture, vehicles, buildings, etc. Such a classification is useful in both pre-mapped i.e., known environments and unknown environments since it increases the potential of the system to explore its environment and navigate.

1. Raw Data sensing: LiDAR is the primary sensor due to its accuracy of detection and also the higher resolution of data and it is effective in providing the shape of the objects in the environment that may contain hazardous obstacles to the vehicle. A stereo vision sensor can provide depth information in addition to the LiDAR. The benefit of using this combination is the accuracy, speed, and resolution of the LiDAR and the quality and richness of data from the stereo vision camera. Together, these two sensors provide an accurate, rich, and fast data set for the object detection layer [18,28,29].
  In a recent study in 2019, Rövid et al. went a step further to utilize the raw data and fuse it to realize the benefits early on in the cycle [30]. They fused camera image data with LiDAR pointclouds closest to the raw level of data extraction and its abstraction.
2. Object Detection: Object Detection is the method of locating an object of interest in the sensor output. LiDAR data scan objects differently in their environment than a camera. Hence, the methodology to detect objects in the data from these sensors would be different as well. The research community has used this technique to detect objects in aerial, ground, and underwater environments [30,31,32,33,34].
3. Object Classification: The Objects are detected and then they are classified into several types so that they can be grouped into small, medium, and large objects, or hazard levels of nonhazardous or hazardous, such that the right navigation can be handled for the appropriate object. Chavez-Garcia et al. [35] fuse multiple sensors including camera and LiDAR to classify and track moving objects.
4. Data Fusion: After the classification, the data are fused to finalize information as input to the control layer. The data fusion layer output will provide location information of the objects in the map of the environment, so that the autonomous vehicle can, for instance, avoid the obstacle or stop if the object is a destination or wait for a state to be reached for further action if the object is deemed a marker or milestone. The control segment will take the necessary action, depending on the behavior as sensed by the sensor suite [18,28,29,35,36,37].

### 2.2. Multiple Sensors vs. Single Sensor

It is a known fact that most of the autonomous systems require multiple sensors to function optimally. However, why should we use multiple sensors? Individual usage of any sensor could impact the system where they are used, due to the limitations in each of those sensors. Hence, to get acceptable results, one may utilize a suite of different sensors and utilize the benefits of each of them. The diversity offered by the suite of sensors contributes positively to the sensed data perception [38,39]. Another reason could be the system failure risk due to the failure of that single sensor [21,27,40] and hence one should introduce a level of redundancy. For instance, while executing the obstacle avoidance module, if the camera is the only installed sensor and it fails, it could be catastrophic. However, if it has an additional camera or LiDAR, it can navigate itself to a safe place after successfully avoiding the obstacle, if such logic is built-in for that failure. Roggen et al., Luo et al., and Foo et al. [41,42,43] performed a study on high-level decision data fusion and concluded that using multiple sensors with data fusion is better than individual sensors without data fusion. In addition to the above, several researchers [27,39,44,45,46] discovered that every sensor used provides a different type, sometimes unique type of information in the selected environment, which includes the tracked object, avoided object, the autonomous vehicle itself, the world it is being used, and so on and so forth, and the information is provided with differing accuracy and differing details.

There are some disadvantages while using multiple sensors and one of them is that they have additional levels of complexity; however, using an optimal technique for fusing the data can mitigate this challenge efficiently. When data are optimally combined, information from different views of the environment gives an accurate model of the environment the system is being used in.

The second was highlighted by Brooks et al. [47] who state:

*A man with one clock knows what time it is. A man with two clocks is never sure!*

That is, there may be the presence of a level of uncertainty in the functioning, accuracy, and appropriateness of the sensed raw data. Due to these challenges, the system must be able to diagnose accurately when a failure occurs and ensure that the failed component(s) are identified for apt mitigation. At a high level, we can term two types of sensor fusion: *Homogeneous data fusion and Heterogeneous data fusion*. As the name states, homogeneous data fusion comprises sensor data of the same types of sensors; there may or may not be the same make or model—for example, a stereo vision camera only, GPS data only, or LiDAR data only, etc. On the other hand, heterogeneous data fusion will have varied sensor data. There could be a suite of sensors like GPS, LiDAR, stereo vision camera or GPS and LiDAR or IMU and GPS, etc. In addition, it must be able to tolerate small differences between the same-sensor readings and be able to merge their small discrepancies into a single sensor reading that is reliable. This is done through data fusion, which we will address later. As an example, let us consider humans; redundancy is built into us, which is we have five different senses and among these senses, we have two eyes and two ears, and an entire body of skin that can sense. We use these senses subconsciously, i.e., without specifically instructing our brains to use them appropriately. This should be implemented purposefully, specifically and carefully into an autonomous system. The above-mentioned researchers [40,47] state that the information obtained by the intelligent system using a single sensor will tend to be incomplete and sometimes inaccurate, due to its inherent limitations and uncertainty.

**Figure 2:**
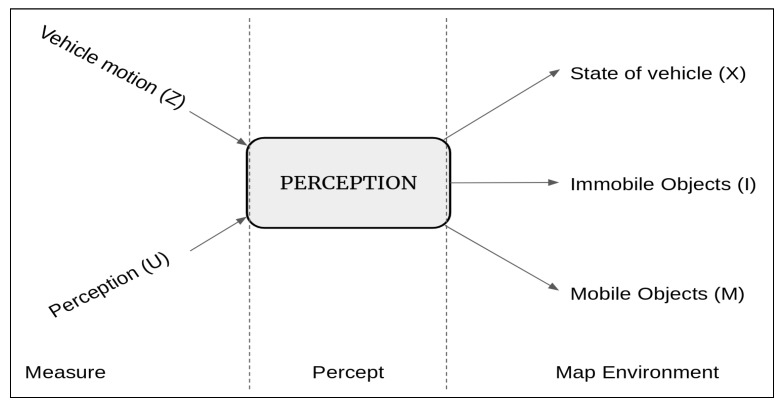
Concepts of perception.

Consider a graphical representation of a simple perception system as given in Figure 2. The system takes in as input, the sensor data of the perception sensors like LiDAR, sonar, camera, etc., and motion sensors like the odometric, navigational sensors, etc. The output comprises location, distance of the objects in the vicinity, and the current state of the robot to name a few. Although these outputs seem similar, details clearly state that they vary in many ways; for example, a vehicular motion sensor will not provide information about obstacles in front of the robot; a camera cannot provide details about the robot’s location like latitude and longitude, etc. (unless a GPS is built into the camera); and therefore a single sensor will not be able to provide all the information that is necessary to optimally perform the complete suite of tasks. Hence, we have the need to use multiple sensors that may be redundant but are complementary and can provide the information to the perception module in the intelligent system. Therefore, the perception module uses information from sensors like LiDAR, camera, sonar, etc. We will detail these sensors and the above-mentioned tasks in the following sections. Combining information from several sensors is a challenging problem [39,48,49].

Rao et al. [29] provide metrics comparing the difference(s) between single sensor and multi-sensors. They state that, if the distribution function depicting measurement errors of one sensor is precisely known, an optimal fusion process can be developed, and this fusion process performs similar to if not better than a single sensor. Users can be reassured that the fused data is better than that of a single sensor. Since the sensing layer is better now, the control application can be standardized independently.

### 2.3. Need for Sensor Data Fusion

Some of the limitations of single sensor unit systems are as follows:

1. Deprivation: If a sensor stops functioning, the system where it was incorporated in will have a loss of perception.
2. Uncertainty: Inaccuracies arise when features are missing, due to ambiguities or when all required aspects cannot be measured
3. Imprecision: The sensor measurements will not be precise and will not be accurate.
4. Limited temporal coverage: There are initialization/setup time to reach a sensor’s maximum performance and transmit a measurement, hence limiting the frequency of the maximum measurements.
5. Limited spatial coverage: Normally, an individual sensor will cover only a limited region of the entire environment—for example, a reading from an ambient thermometer on a drone provides an estimation of the temperature near the thermometer and may fail to correctly render the average temperature in the entire environment.

The problems stated above can be mitigated by using a suite of sensors, either homogeneous or heterogeneous [38,44,46,50,51] in addition to mitigating the issues of the above data fusion. Some of the advantages of using multiple sensors or a sensor suite are as follows:

1. Extended Spatial Coverage: Multiple sensors can measure across a wider range of space and sense where a single sensor cannot
2. Extended Temporal Coverage: Time-based coverage increases while using multiple sensors
3. Improved resolution: A union of multiple independent measurements of the same property, the resolution is better, i.e., more than that of single sensor measurement.
4. Reduced Uncertainty: As a whole, when we consider the entire sensor suite, the uncertainty decreases, since the combined information reduces the set of unambiguous interpretations of the sensed value.
5. Increased robustness against interference: An increase in the dimensionality of the sensor space (measuring using a LiDAR and stereo vision cameras), the system becomes less vulnerable against interference.
6. Increased robustness: The redundancy that is provided due to the multiple sensors provides more robustness, even when there is a partial failure due to one of the sensors being down.
7. Increased reliability: Due to the increased robustness, the system becomes more reliable.
8. Increased confidence: When the same domain or property is measured by multiple sensors, one sensor can confirm the accuracy of other sensors; this can be attributed to re-verification and hence the confidence is better.
9. Reduced complexity: The output of multiple sensor fusion is better; it has lesser uncertainty, is less noisy, and complete.

### 2.4. Levels of Data Fusion Application

Data fusion can be applied at various levels of data gathering or data grouping and are dependent on the abstraction levels of data. We will see in the upcoming sections the abstraction levels of data fusions. The abstraction levels of data fusion are:

1. *Decision or High-level data fusion*. At the highest level, the system decides the major tasks and takes decisions based on the fusion of information, which is input from the system features [41,43].
2. *Feature or mid-level data fusion*. At the feature level, feature maps containing lines, corners, edges, textures, and lines are integrated and decisions made for tasks like obstacle detection, object recognition, etc. [52,53,54].
3. *Raw-data or low-level data fusion*. At this most basic or lowest level, better or improved data are obtained by integrating raw data directly from multiple sensors; such data can be used in tasks. This new combined raw data will contain more information than the individual sensor data. We have summarized the most common data fusion techniques and the benefits of using that technique as well [55].

The versatility involved in the implementation of data fusion can be realized by the above levels of application.

### 2.5. Data Fusion Techniques

Nature provides us sensing as one of its most important methods for survival in the animal or plant kingdom. In the animal kingdom, this can be seen as a seamless integration of data from various sources, some overlapping and some non-overlapping to output information which is reliable and feature-rich that can be used in fulfilling goals. In nature, this capability is most essential for survival, to hunt for food or to escape from being hunted. As an example in wildlife, consider bears and compare their sensory capabilities; they have a sharp color close-up vision but do not have a good long distant vision [56]. However, their hearing is excellent because they have the capability to hear in all directions. Their sense of smell is extremely good. They use their paws very dexterously to manipulate wide-ranging objects, from picking little blueberries to lifting huge rocks. Often, bears touch objects with their lips, noses, and tongue to feel them. Hence, we can surmise that their sense of touch is very good. Surely they combine signals from the five body senses i.e., sound, sight, smell, taste, and touch) with information of the environment they are in, and create and maintain a dynamic model of the world. At the time of need, for instance, when a predator is around, it prepares itself and takes decisions regarding the current and future actions [56]. Over the years, scientists and engineers have applied concepts of such fusion into technical areas and have developed new disciplines and technologies that span over several fields. They have developed systems with multiple sensors and devised mechanisms and techniques to augment the data from all the sensors and get the ’best’ data as output from this set of sensors, also known as a ’suite of sensors’. In short, this augmentation or integration of data from multiple sensors can simply be termed as multi-sensor data fusion.

Kanade et al. in the early 1980s used aerial sensor data to obtain passive sensor fusion of stereo vision imagery. Crowley et al. performed fundamental research in the area of data fusion, perception, and world model development that is vital for robot navigation [57,58,59]. They realized that data fusion needs to be applied incrementally in their perception problem [59]. They developed similar techniques [58] that used Kanade’s incremental approach to build a world model for robot navigation. They generalized fusion work and documented that, using cyclical processes, one can achieve good perception. Brooks developed a visual ad-hoc technique [60] that was used in robot perception.

Bayesian estimation theory was recommended by Smith et al. for robotic vision [61]. Whyte documented in his research thesis the derivation techniques for optimizing and integrating sensor information, that may be considered as extensions of estimation theory [62]. It was also implemented in a recent study about system noise [63]. Faugeras et al. performed stereo vision calibration using an adaptation of estimation theory as well [64].

The community witnessed a growth in the development of techniques that performed the minimization of a required energy function which provided quantitative measurements and constraints and calculates how much the measurements and constraints are violated [65,66]. Further research was performed by Koch et al. [67,68], Blake [69], and so on, in the areas of implementing neural networks to implement regularization algorithms for the data fusion. Reinforcement learning networks were implemented to implement multisensor data fusion [70].

Symbolic reasoning techniques using artificial intelligence and machine learning contributed to rule-based inference which was studied in OPS5 [71,72], MYCIN [73], and BBI [74]. Any of these inference techniques coupled with constraint-based reasoning techniques.

Over the years, several techniques that have emerged as data fusion paradigms are Zadeh’s fuzzy logic [75], Duda’s symbolic uncertainty management [76], and Shafer’s combined evidence techniques that give a basis for inference under uncertainty [77]

Crowley et al. provide a set of numerical techniques that are represented by a primitive comprising a vector of property estimates and their respective precisions. They showed that Kalman filter prediction equations provide a means for prediction of the model’s state [57].

Waltz et al. [44] and Llinas and Hall [78] define the term *multisensor data fusion* as a technology concerned with combining data from multiple (and possibly diverse) sensors to make inferences about a physical environment, event, activity, or situation.

The International Society of Information Fusion defines information fusion as [79]

“*Information Fusion encompasses theory, techniques, and tools conceived and employed for exploiting the synergy in the information acquired from multiple sources (sensor, databases, information gathered by human, etc.) such that the resulting decision or action is in some sense better (qualitatively or quantitatively, in terms of accuracy, robustness, etc.) than would be possible if any of these sources were used individually without such synergy exploitation*.”

The definition of multi-sensor data fusion by Waltz and Llinas [44] and Hall [24] is given as:

The technology concerned with the combination of how to combine data from multiple (and possible diverse) sensors to make inferences about a physical event, activity, or situation

The definition, process, and one of the purposes of data fusion is elicited by Elmenreich et al. [80] as:

“*Sensor Fusion is the combining of sensory data or data derived from sensory data such that the resulting information is in some sense better than would be possible when these sources were used individually*”.

With respect to the output data types of the sensors, we can broadly categorize them into homogeneous sensor data and heterogeneous sensor data. Heterogeneous sensor data comprise of different types of sensing equipment, like imaging, laser, auditory, EEG, etc. For example, a monocular camera (RGB) will have pure image data, while a stereo vision camera (RGB-D) could have imaging data for both the cameras and a depth cloud for the depth information, an EEG could output signal details and LiDAR outputs’ location details of the object of interest with respect to the LiDAR. Systems with multi-sensor fusion are capable of providing many benefits when compared with single sensor systems. This is because all sensors suffer from some form of limitation, which could lead to the overall malfunction or limited functionality in the control system where it is incorporated.

Garcia et al. in 2017 proposed a novel sensor data fusion methodology in which the augmented environment information is provided to the intelligent vehicles with LiDAR, camera, and GPS. They propose that their methodology leads to safer roads by data fusion techniques in single-lane carriage-ways where casualties are higher than in other road types. They rely on the speed and accuracy of the LiDAR for obstacle detection and camera-based identification techniques and advanced tracking and data association algorithms like Unscented Kalman Filter and Joint Probabilistic Data Association [81]. Jahromi et al. proposed a real-time hybrid data fusion technique in 2019 [82]. Extended Kalman Filter (EKF) based nonlinear state estimation and encoder–decoder based Fully Convolutional Neural Network (FCNN) are used on a suite of camera, LiDAR, and radar sensors. Data fusion is a vast area with numerous techniques; we provide advantages and disadvantages of data grouping/association, state estimations, and distributed systems [29,83,84,85]. The following subsections highlight some of the algorithms used in data fusion.

#### 2.5.1. K-Means

K-Means is a popular algorithm that has been widely employed;

Some prominent advantages are:

- Simpler to implement compared to other techniques
- Good generalization to clusters of various shapes and sizes, such as elliptical clusters, circular, etc.
- Simpler and easy adaption to new examples
- Convergence is guaranteed.
- Scales to large data sets
- Centroid position can be warm-started

Some prominent disadvantages:

- Optimal solution for the cluster centers are not always found by the algorithm;
- The algorithm assumes that the covariance of the dataset is irrelevant or that it has been normalized already.
- The system must have knowledge of the number of clusters a priori.
- Assumption is made that this number is optimum.

#### 2.5.2. Probabilistic Data Association (PDA)

PDA was proposed by Bar-Shalom and Tse, and it is also known by “modified filter of all neighbors” [86]. The functionality is to assign an association probability to each hypothesis from the correct measurement of a destination/target and then process it.

Prominent advantages are:

- Tracking target excellence: Excellent for tracking targets that do not make sudden changes in their navigation
  PDA is mainly good for tracking targets that do not make abrupt changes in their movement pattern. The prominent disadvantages are [86,87]:

- Track loss: PDA might display poor performance when the targets are close to each other because it ignores the interference with other targets and hence there is a possibility that it could wrongly classify the closest tracks.
- Suboptimal Bayesian approximation: PDA gives suboptimal Bayesian approximation when the source of information is uncertain; this might be seen when a LiDAR scans a pole.
- One target: PDA gives incorrect results in the presence of multiple targets since the false alarm model does not work well. The Poisson distribution typically models the number of false, with an assumption of uniform distribution
- Track management: Problems of tracking algorithms must be provided for track initialization and track deletion since PDA needs this a priori.

#### 2.5.3. Joint Probabilistic Data Association

The prominent advantages are as follows [87,88,89]:

- Robust: JPDA is robust compared to PDA and MHT.
- Multiple object tracking: The algorithm can be used to track multiple agents (however, with a caveat)
- Representation of multimodal data: Can represent multimodal state densities, which represent the increase in robustness of the underlying state estimation process

The prominent disadvantages of JPDA are as follows [87,88,89]:

- Computationally expensive: JPDA is a computationally expensive algorithm when employed in multiple target environments since the number of hypotheses’ increments exponentially with the number of targets.
- Exclusive mechanism: It requires an exclusive mechanism for track initialization.

#### 2.5.4. Distributed Multiple Hypothesis Test

The main advantages of MHT-D are [90]:

- Very useful in distributed and de-centralized systems
- Outperforms JPDA for the lower densities of false positives
- Efficient at tracking multiple targets in cluttered environments
- Functions also as an estimation and tracking technique

The main disadvantage of the MHT-D is as follows [90]:

- Exponential computational costs that are in the order of $O(n^{X})$, where *X* is the number of variables to be estimated and *n* is the number of possible associations

Another type of fusion technique is by *state estimation*.

#### 2.5.5. State Estimation

Also known as tracking techniques, they assist with calculating the moving target’s state, when measurements are given [87]. These measurements are obtained using the sensors. This is a fairly common technique in data fusion mainly for two reasons: (1) measurements are usually obtained from multiple sensors; and there could be noise in the measurements. Some examples are Kalman Filters, Extended Kalman Filters, Particle Filters, etc. [91]. We discuss state estimation techniques in Section 3.5.

#### 2.5.6. Covariance Consistency Methods

These methods were proposed initially by Uhlmann et al. [84,87]. This is a distributed technique that maintains covariance estimations and means in a distributed system. They comprise of estimation-fusion techniques.

Some prominent advantages are:

- Efficient in distributed systems; i.e., multimodal multi-sensors as well
- Fault-tolerant for covariance means and estimates

Some prominent disadvantages are:

- If the Kalman filter is used for estimation, the exact cross-covariance information must be determined. This could pose a big challenge.
- Suboptimal results are realized if the iterative application of the technique is used to process a sequence of estimates for a batch application for simultaneous fusion of the estimates.

#### 2.5.7. Decision Fusion Techniques

These techniques can be used when successful target detection occurs [87,92,93]. They enable high-level inference for such events.

Some prominent advantages are:

- Enables the user to arrive at a single decision from a set of multiple classifiers or decision-makers
- Provides compensatory advantage for other sensors when one sensor is deficient, in a multi-sensor system
- Enables a user to adjust the decision rules to arrive at the optimum.

Some prominent disadvantages are:

- Establishing a priori probabilities is difficult
- When a substantial number of events that depend on the multiple hypotheses occur, this will be very complex and a hypothesis must be mutually exclusive
- Decision uncertainty is difficult to finalize

#### 2.5.8. Distributed Data Fusion

As the name suggests, this is a distributed fusion system and is often used in multi-agent systems, multisensor systems, and multimodal systems [84,94,95].

Some prominent advantages are:

- Enables usage across dynamic and distributed systems
- Communication costs can be low since systems can communicate with each other after onboard processing at the individual agents/nodes

Some prominent disadvantages are:

- Spatial and temporal information alignment
- Out-of-sequence measurements
- Data correlation challenges
- Systems may need robust communication systems to share information.

### 2.6. Classifications of Data Fusion Techniques

Classification of data fusion is fuzzy and fluid, in that it is quite tedious and complex to follow and adhere to strict processes and methodologies. Many criteria can be used for the classification of data fusion. Castanedo discussed [87] the techniques and algorithms for state estimation, data association and finally a higher-level decision fusion. Foo performed a study of high-level data fusion in tactical systems, biomedical systems, information science and security, disaster management, fault detection, and diagnosis [43]. Dasarathy et al. [96] discuss data fusion methods and several techniques. Luo et al. [38] discuss abstraction levels and Steinberg et al. via JDL [97] perform basic research in data fusion. The subsections below provide a brief introduction on how we can classify data fusion. Some of these techniques are given in Table 1.

#### 2.6.1. Data Type of Sensor Input and Output Values

Several types of classification emerged out of Dasarathy’s input–output data fusion [96]. They can be summarized as follows: *Data-in-Data-out (DAI-DAO)*: Raw data are input and raw data are extracted out. *Data-in-Feature-out (DAI-FEO)*: Raw data are sourced, but the system provides features extracted out of the data as output. *Feature-in: Feature-out (FEI-FEO)*: Features from previous steps of fusion or other processes are fed into the fusion system and better features or higher-level features are output. New and improved features are output as part of this type of fusion. This is also called Feature-fusion [96]. *Feature-in: Decision-out (FEI-DEO)*: The features fed into the input system as the source are processed to provide decisions for tasks and goals as output. This is where simple or high-level features are accepted as input, and processed and decisions are extracted for the system to follow. Most of the present-day fusion is of this type of classification technique. *Decision-in-Decision-out (DEI-DEO)*: Simple and lower-level decisions are accepted by the system and higher-level better decisions are processed out. This is a type of fusion is also called Decision-fusion [96].

#### 2.6.2. Abstraction Levels

In a typical perception system, one comes across the following abstraction of data: pixel, signal, symbols, feature-characteristics [38]. 

*Pixel level classification*: is performed on image input from sensors like monocular, stereo vision, or depth cameras, IR cameras, etc. to a system; image processing that is used to improve tasks that look for and extract objects; object features use this technique.

*Signal level classification*: is performed on data involving signals from sensors like LiDAR, sonar, audio, etc. The signal data are directly operated on and output rendered.

*Symbol level classification*: is a technique that employs methods to represent information as symbols. This is similar to the decision-fusion technique of Dasarathy [96] and called decision level.

*Characteristic level classification*: extracts features from signals or images while processing the data and is called feature level.

#### 2.6.3. JDL Levels

Data fusion models divided into five processing layers, interconnected by a data bus to a relationship database [97,98]

*Layer 0:* Processes source data comprised of pixel and signal. Information is extracted, processed, reduced, and output to higher layers.
*Layer 1:* Data output from layer 0 is processed here and refined. Typical processes are alignment in the spatial-temporal information, correlation, clustering, association and grouping techniques, false-positive removal and reduction, state estimation, image feature data combination, and state estimations. Classification and identification: state and orientation are the typical outputs. It also performs input data transformation to obtain consistent and robust data-structures.
*Layer 2:* Based on other output of layer 1 or the object refinement layer, analysis of the situation is performed. Based on the data input and the present and past decisions, the situation assessment is performed. A set of high-level inferences is the outcome of this layer. Identification of events and activities are performed.
*Layer 3:* The output of layer 2 i.e., the significant activities and current events are assessed for impact on the system. Prediction of an outcome and threat analysis is performed at this layer.
*Layer 4:* Overall processes from layer 0 through layer 3 are optimized and improved. Resource control and management, task scheduling, and prioritizing are performed to make improvements.

#### 2.6.4. Data Source Relationships

This type of classification uses concepts of data redundancy, data complementing, and data combination [87]. Video data overlaps can be called redundant data sources and can be optimized. This is the area of data source classification wherein the same destination or target is identified by multiple data sources. Complementary data sources provide different inputs that can be combined to form a complete target or scene or object—for example, a complete scene if formed using different cameras and the scene can be put together from individual pieces. Combining data sources in a cooperative environment gives a result that is more complex than the input source information.

#### 2.6.5. System Architecture

This type of classification is based on the architecture of the data fusion system. The architecture could be hierarchical, distributed or decentralized, centralized, etc. [85,87,96]. This prompts us to think that the researchers classified these systems based on how many agents/nodes are available, how the sensors are spread across these agents/nodes. In a decentralized architecture, all the agents take part in the data fusion task. Each system processes its own and its neighbor’s data. The advantages are processing faster since each system could be processing smaller chunks of data. The cons of this process are the high communication costs since several systems need to communicate with each other and the cost is $\omega {\left(n\right)}^{2}$, at each step of communication, and *n* is the number of nodes. The process is costliest if each node has to communicate with every one of its peers. Contrary to this, in a centralized architecture, a powerful single system will perform the data fusion. Suboptimal systems could end up being resource hogs that take up a lot of resources in the form of bandwidth since raw data are transferred from the sensors to the central processing system. When a higher number of sensors are used, this type of architecture will pose huge resource issues. Moreover, the central unit would need to be very powerful to process and perform data fusion, which could mean an expensive system.

*Distributed or decentralized systems:* State estimation and data processing are performed locally and then communicated to the other systems. Single node to groups of systems form the range of processing in this architecture. The fusion node processes the result only after the individual data processing at the local level is completed [94,99,100].
*Hierarchical systems:* A system architecture, wherein the higher-level nodes control the lower-level nodes and a mechanism of hierarchical control of data fusion is set up, is the hierarchical data fusion system. In this type of architecture, a combination of distributed decentralized nodes could be employed to achieve data fusion. Back in the second half of the 1990s, Bowman et al. proposed a hierarchical data fusion system [101] which was reviewed by Hall et al. [21]. Taropa et al. in 2006 proposed a hierarchical data fusion model [102] in which they use real-time objects in a highly flexible framework and provide these features through an API. Dieterle et al. proposed a data fusion system for object tracking [103]. In the publication, they combine sensor information using a hierarchical data fusion approach and show that this approach drastically improves robustness in object detection with respect to sensor failures and occlusions.

## 3. Sensor Hardware

We will now briefly introduce some of the hardware that could be used for data fusion in vehicular navigation.

### 3.1. LiDAR

Light Detection and Ranging (LiDAR) is a technology that is used in several autonomous tasks and functions as follows: an area is illuminated by a light source. The light is scattered by the objects in that scene and is detected by a photo-detector. The LiDAR can provide the distance to the object by measuring the time it takes for the light to travel to the object and back.

NOAA states:

*LIDAR, which stands for Light Detection and Ranging, is a remote sensing method that uses light in the form of a pulsed laser to measure ranges (variable distances) to the Earth. These light pulses—combined with other data recorded by the airborne system—generate precise, three-dimensional information about the shape of the Earth and its surface characteristics* [104].

#### 3.1.1. Data Generation in a LiDAR

Different types of data are generated by a LiDAR. Some are highlighted below.

1. Number of Returns: The Light pulses from a LiDAR can penetrate a canopy in a forest. This also means that LiDAR can hit the bare Earth or short vegetation.
2. Digital Elevation Models: Digital Elevation Models (DEM) are earth (topographic) models of the Earth’s surface. A DEM can be built by using only ground returns. This is different from Digital Terrain Models (DTM), wherein contours are incorporated.
3. Digital Surface Models: A Digital Surface Model (DSM) incorporates elevations from man-made and natural surfaces. For example, the addition of elevation from buildings, tree canopies, vehicular traffic, powerlines, vineyards, and other features.
4. Canopy Height Model: Canopy Height Models (CHM) provides the true height of topographic features on the ground. This is also called a Normalized Digital Surface Model (nDSM).
5. Light Intensity: Reflectivity or Light intensity varies with the composition of the object reflecting the LiDAR’s return. Light intensity is defined as the reflective percentages.

#### 3.1.2. Classifying the LiDAR

LiDAR can be generally classified based on the data returned, technology used, area of usage [105].

- **Data Returned by the LiDAR**: LiDAR types based on storing the data returned from the object [106]:
  1. Discrete LiDAR: While scanning, the data returned are in the form of 1st, 2nd, and 3rd returns, due to the light hitting multiple surfaces. Finally, a large-final pulse is returned. This can be seen when a LiDAR hits a forest canopy [107]. When the LiDAR stores the return data individually/discretely, it takes each peak and separates each return.
  2. Continuous/Full waveform LiDAR: When the entire waveform is saved as one unit, its a continuous or full form LiDAR [108]. A lot of LiDARs use this form of recording.
- **Lidar types based on technology**: The following technology types can be considered as well while classifying LiDARs [105,109]
  1. Mechanical-scanners: Macro-scanners, Risley prisms, Micro-motion.
  2. Non-Mechanical-scanners: MEMS, Optical phased arrays, electro-optical, liquid crystal.
  3. Flash-LiDAR-non-scanners
  4. Structured light-non-scanners
  5. Multicamera-stereo-non-scanners
- **Based on area of usage**: Two types of LiDAR broadly used are: topographic and bathymetric [104]. Topographic LiDARs are typically used in land mapping, and they use near-infrared laser and bathymetric LiDARs use green light technology for water-penetration to measure river bed elevations and seafloor.
  In *Topographic LiDAR*, the two main types are 2D (single scan) and 3D (multiple scan). Some brands of topographic LiDAR are Velodyne [110], another model from Velodyne, the HDL-64E provides a 3D laser scan i.e., 360° horizontal and 26.9° vertical field of view (FOV), while 2D LiDARs like the TiM571 LiDAR scanning range finder from SICK provide a 2D 220° FOV this is very similar to RPLidar [111] from Slamtech, Ouster [112] from Ouster laser scanners, Eclipse mapping systems [113]. The *Bathymetric LiDARs* use the green spectrum technology and are predominantly used for water surface and underwater mapping tasks. A small listing and background of Bathymetric LiDARs are given by Quadros et al. from Quadros [114]. However, bathymetric LiDARs are out of the scope of this survey due to its nature of use.

#### 3.1.3. Advantages and Disadvantages in Using LiDAR

LiDARs are very useful in detecting objects and developing an environment model [93,104,114]. It does have both usage advantages and disadvantages. Advantages include Safety in usage, fast scans of the environment, high accuracy, and some can capture data even at 2500 m and have better resolution compared to other scan systems like Radar.

Disadvantages include: Many products are still very expensive, data are not as rich as an RGB camera with a good resolution, a single data point may not be accurate and high volume data points will need to be used, their scans and eventual point clouds are too big and consume a lot of space, and 2D LiDARs are useful mainly as line scanners and hence are sparingly used.

### 3.2. Camera

The types of camera are Conventional color cameras like USB/web camera; RGB, RGB-mono, and RGB cameras with depth information; RGB-Depth (RGB-D), 360° camera, and Time-of-Flight (TOF) camera.

#### 3.2.1. RGB Family of Camera

An RGB camera is typically a camera equipped with a standard CMOS sensor through which the colored images of the world are acquired. The acquisition of static photos is usually expressed in megapixels [115].

Advantages and disadvantages of RGB cameras are as follows:

Advantages include availability of several inexpensive cameras, and they do not need any specialized drivers, simplicity in usage, etc.

The disadvantages include that the presence of good lighting is essential, some of the high-end cameras that have great resolution are very expensive, and there are RGB-D cameras that cannot efficiently capture surfaces that are reflective, absorptive, and transparent such as glass and plastic.

#### 3.2.2. 360° Camera

A 360° camera captures dual images or video files from dual lenses with 180° field of view and either performs an on-camera automatic stitch of the images/video or lets the user perform off-board stitching of the images, to give a full 360° view of the world [28,116,117,118].

Some advantages and disadvantages are as follows:

Advantages include new technology possibilities in usage and improvements being higher, and hardware or software may be used to get 360 images, etc.

Disadvantages include diminished quality, few cameras are expensive, long rendering time, storage may be needed more in high-resolution cameras, etc.

#### 3.2.3. Time-of-Flight (TOF)

The TOF gives depth information based on IR and camera technology. It works by emitting an infrared light signal and measures how long the signal takes to return and calculates the depth based on extracted data. This information can be used with several navigation-related modules like mapping and obstacle avoidance [119,120,121].

Some advantages and disadvantages are highlighted in [122] as follows:

Advantages include high speed, efficient usage of computation since TOF uses a one look approach compared to the multiple scans of laser scanners, long working distance, depth information up to 5 m given in real-time, wide application range (feature-filled or featureless, depth information given by camera in the presence or absence of ambient light).

Disadvantages include low resolution, relatively high power consumption due to which high heat may be generated, affected by object’s reflective, color and complexity of the environment, may need additional management of subjects’ background lighting, multiple path reflections, usage of multiple TOF at the same time may have interference with each other, supported application scenarios are less, and development and support groups are low in number.

In some autonomous vehicles, radar is used in addition to camera [123,124] (however, the study of radar is out of the scope of this paper)

### 3.3. Implementation of Data Fusion with the Given Hardware

We review an input–output type of the fusion as described by Dasarathy et al. [96]. They propose a classification strategy based on input–output of entities like data, architecture, features, and decisions. The fusion of raw data in the first layer, a fusion of features in the second, and finally the decision layer fusion. In the case of the LiDAR and camera data fusion, two distinct steps effectively integrate/fuse the data [28,117,125]:

1. Geometric alignment of the sensor data
2. Resolution match between the sensor data

Let us review these two steps in greater detail.

#### 3.3.1. Geometric Alignment of the Sensor Data

The first and foremost step in the data fusion methodology is the alignment of the sensor data. In this step, the logic finds LiDAR data points for each of the pixel data points from the optical image. This ensures the geometric alignment of the two sensors [28].

#### 3.3.2. Resolution Match between the Sensor Data

**Figure 3:**
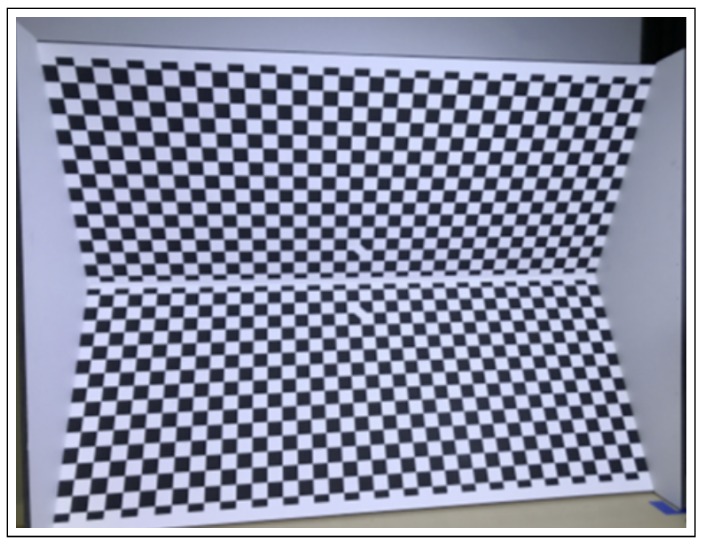
Depth calibration [127].

Once the data is geometrically aligned, there must be a match in the resolution between the sensor data of the two sensors. The optical camera has the highest resolution of 1920 × 1080 at 30 fps, followed by the depth camera output that has a resolution of 1280 × 720 pixels at 90 fps and finally the LiDAR data have the lowest resolution. This step performs an extrinsic calibration of the data. Madden et al. performed a sensor alignment [126] of a LiDAR and 3D depth camera using a probabilistic approach. De Silva et al. [28] performed a resolution match by finding a distance value for the image pixels for which there is no distance value. They solve this as a missing value prediction problem, which is based on regression. They formulate the missing data values using the relationship between the measured data point values by using a multi-modal technique called Gaussian Process Regression (GPR), developed by Lahat et al. [39]. The resolution matching of two different sensors can be performed through extrinsic sensor calibration. Considering the depth information of a liDAR and the stereo vision camera, 3D depth boards can be developed out of simple 2D images. Figure 3 shows the depth calibration board. The dimensions of this board are: length 58${}^{\prime \prime }\times$ width 18${}^{\prime \prime }\times$ height 41.5${}^{\prime \prime }$.

**Figure 4:**
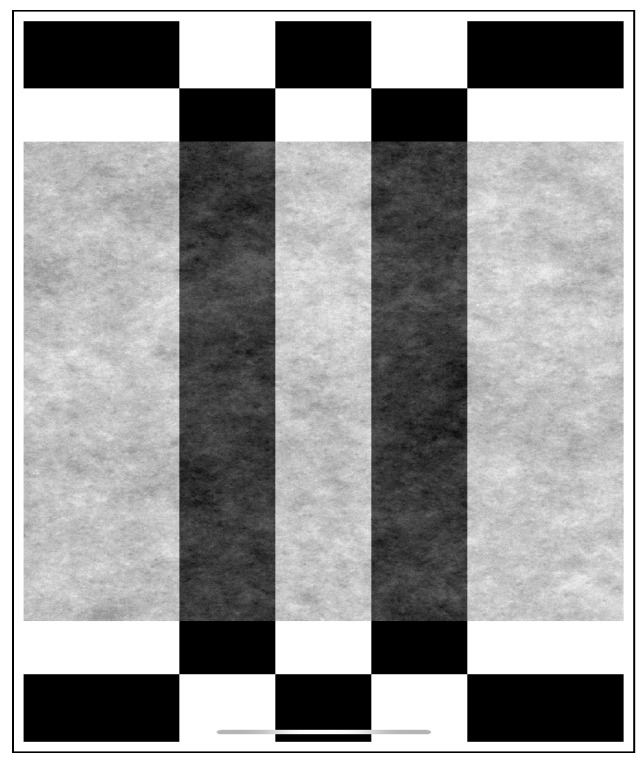
Realsense phone calibration tool [127].

**Figure 5:**
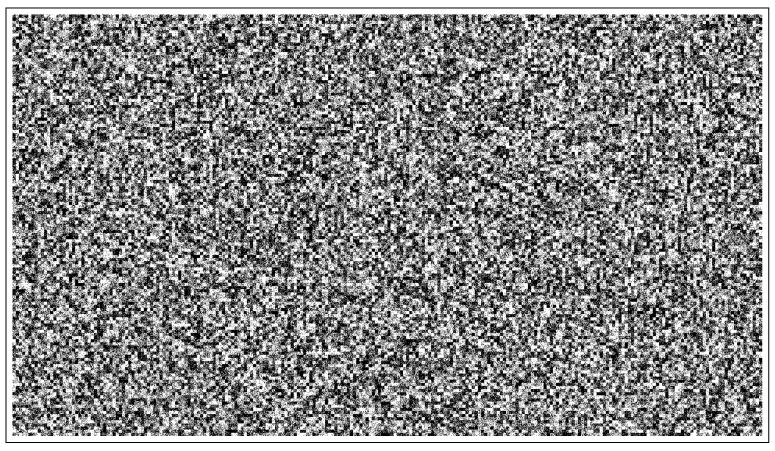
Realsense iPhone speck pattern for calibration [128].

For a stereo vision or depth camera like the Intel Realsense d435, there is a need to perform a depth scale calibration. Figure 4 shows the phone calibration tool [127]. Another addition to the calibration toolkit is the speck pattern board. These pattern boards in (not to scale) Figure 5 give us better results since there is a higher spatial frequency content with limited or no laser speckle. It has been documented that a passive target or LED-based projector gives about 25–30% better depth accuracy than a laser-based projector [127]. After using adequate turning mechanisms, the depth accuracy can be improved even more. The projector can be a drawback in some cases, and it may help to turn off the projection from the camera and light up the subject using clean white light [128]. It is also observed that the RealSense cameras have better performance in open bright sunlight since there is better visibility of the natural textures. It should be noted that, in the case of the depth cameras, the stereo vision has a limitation due to the quality differences between the left and right images.

There are several calibration techniques for the LiDAR and camera, wherein Mirzaei et al. [129] have provided techniques for intrinsic calibration of a LiDAR and extrinsic calibration based on camera readings.

Dong et al. [130] have provided a technique for extrinsic calibration of a 2D LiDAR and camera. Li et al. [131] also have developed a technique for 2D LiDAR and camera calibration—however for an indoor environment. Kaess et al. [132] developed a novel technique to calibrate a 3D LiDAR and camera.

### 3.4. *Challenges with Sensor Data Fusion*

Several challenges have been observed while implementing multisensor data fusion. Some of them could be data related to like: complexity in data, conflicting and/or contradicting data, or they can be technical such as resolution differences between the sensors, the difference in alignment between the sensors [28], etc. We review two of the fundamental challenges surrounding sensor data fusion, which are the resolution differences in the heterogeneous sensors and understanding and utilizing the heterogeneous sensor data streams [28] while accounting for many uncertainties in the sensor data sources [39]. We focus on reviewing the utilization of the fused information in the autonomous navigation, which is challenging since many autonomous systems work in complex environments, be it at home or work, which is to assist persons with severe motor disabilities to handle their navigational requirements and hence pose significant challenges for decision-making due to the safety, efficiency, and accuracy requirements. For reliable operation, decisions on the system need to be made by considering the entire set of multi-modal sensor data they acquire, keeping in mind a complete solution. In addition to this, the decisions need to be made considering the uncertainties associated with both the data acquisition methods and the implemented pre-processing algorithms. Our focus in this review is to survey the data fusion techniques that consider the uncertainty in the fusion algorithm.

Some researchers used mathematical and/or statistical techniques for data fusion. Others used techniques comprised of reinforcement learning in implementing multisensor data fusion [70], where they encountered conflicting data. In this study, they fitted smart mobile systems with sensors that enabled the systems to be sensitive to the environment(s) they were active in. The challenge they try to solve is mapping the multiple streams of raw sensory data Smart agents to their tasks. In their environment, the tasks were different and conflicting, which complicated the problem. This resulted in their system learning to translate the multiple inputs to the appropriate tasks or sequence of system actions.

Brooks et al. [47] achieve sensor data robustness, reliability, and resolve issues like mechanical failures, noise, transient errors using multiple sensors, whose data is fused. They recommend fusing readings from multiple heterogeneous sensors. This made their overall system less sensitive to failures from one technology. Crowel et al. developed mathematical tools to counter uncertainties with fusion and perception [133]. Other implementations include adaptive learning techniques [134], wherein the authors use D-CNN techniques in a multisensor environment for fault diagnostics in planetary gearboxes.

The other challenges are dependent on the sensor itself, i.e., the hardware, or the physics that are used by the hardware. Structural errors in the hardware are an example. These errors are the difference(s) between a sensor’s expected value and measured value, whenever the sensor is used for data collection. Repeated differences can be calculated using a technique called sensor calibration. Before using any sensor, it needs to be calibrated. This will ensure a consistent measurement, i.e., where all the sensors can be fused uniformly.

Broadly, one can differentiate calibration into extrinsic and intrinsic. Extrinsic calibration entails finding external parameters that are used in the sensors—for example, parameter differences between a LiDAR’s alignment/orientation and a camera’s alignment/orientation [130,135]. In another case, it may be the LiDAR’s orientation and location in its working environment or world. In contrast, intrinsic calibration entails finding the differences within the same sensor. For example, relationship(s) between the camera coordinates and its pixel coordinates. Usually, the manufacturer performs intrinsic calibration and communicates the details to the end-user in the user guide/manual.

Researchers have found that extrinsic calibration can be challenging when the number of agents is high as in cases of swarms of robots [129,130,132]. For example, senior living where the swarms of autonomous wheelchairs work together to share information about location, situation awareness, etc.; this could be attributed to the variations that exist between sensors due to manufacturing differences, types of sensors, and autonomous system types. In such an example, the calibration duration will be large if there is a large number of autonomous systems; in fact, it could be exponential and hence exorbitant and unacceptable. Reducing both the time required for the process and the complexity is essential.

### 3.5. Sensor Data Noise

Every sensor has an amount of noise that is inherent to its properties. There have been many attempts at reducing or removing the noise—for instance, in object detection [136] wherein the authors provide a method and technique to remove noise in LiDAR intensity images. They use a type of diffusion filtering called anisotropic filtering to retain the scanned object space details and characteristics. The second research is where the background noise is removed [137], wherein the authors develop a methodology to identify background noise under the clear atmospheric condition and derive equations to calculate the noise levels. Topics other than object detection are speech recognition [138,139]. In this section, we discuss filtering noise using the Kalman Filter. Kalman filter is over five decades old and is one of the most sought after filtering techniques. We will discuss two flavors of Kalman filter, namely: Extended Kalman Filter and Unscented Kalman Filter.

In addition to the sensing information, every sensor is bound to have a level of noise and, while using these sensors, one will soon realize that at least a small amount of noise is bound to exist in addition to measurement and estimation of uncertainties. When such errors or uncertainties occur, it is required to use techniques that mitigate their effects on the system. This now becomes a complex problem of estimating the state(s) of the system after the system becomes observable. Mathematical algorithms that accomplish this are the *filtering techniques*. Filtering techniques are applicable in several domains like economics, science, and engineering. Localization systems can make use of these techniques as there is an innate level of sensor measurement noise and uncertainty with their pose estimation. Filtering techniques have been used in many localization systems and two of the most popular filtering algorithms are Kalman filters and particle filters.

#### 3.5.1. Kalman Filters

Kalman filters (KF) were introduced by Rudolf Kalman in 1960 [140]. It is also known as Linear Quadratic Estimation (LQE) in the field of controls and autonomous systems. KF is versatile and has been extensively used in the areas of autonomous systems, signal processing, system navigation, defense, aerospace, etc., and it is an iterative algorithm that uses Bayesian inference to estimate the probabilistic distribution of the uncertain/unknown variables. They use a series of measurements that have noise from measurements and process(es). This is because unknown variables can be estimated better with multiple measurements than with a single measurement. The algorithm is optimized to run in real-time and needs only the previous system state and the current input measurement. The KF starts with the system model and the known control inputs to that system, and multiple sequential measurements (measurements from sensors) and forms an estimate of the system’s varying quantities (provided in the state matrices). Incidentally, it is found to be better than the estimate obtained using a single measurement. Kalman Filter can also be broadly categorized as a common sensor fusion and data fusion algorithm.

A Dynamic System Model can be represented as follows:

(1) $$x_{k}=Ax_{k-1}+Bu_{k}+w_{k-1},$$

(2) $$z_{k}=Hx_{k}+v_{k},$$

where:

$x_{k}$: Current estimate,
$x_{k-1}$: Estimate of the signal in Previous state,
$u_{k}$: Control signal,
$z_{k}$: Measured value from the sensors,
$w_{k-1}$: Process noise in the previous iteration,
$v_{k}$: Measurement noise in the present iteration.

Equations (1) and (2) are a simple system model where *k* denotes the current time sample.

Equation (1) denotes the current estimate of a state variable $x_{k}$, which is comprised of the previous system state $x_{k-1}$, the control signal $u_{k}$, and the process noise in the previous iteration $w_{k-1}$.

Equation (2) calculates the current measurement value $z_{k}$, which is a linear combination of the unknown variable and the measurement noise $v_{k}$ and this is usually a Gaussian. A, B, and H are matrices that provide the weights of the corresponding component of the equation. These values can be provided a priori and are system dependent. A Gaussian distribution with a zero mean contributes two noise values, namely $w_{k-1}$ and $v_{k}$; these have covariance matrices named Q and R, respectively, and they are estimated a priori, although they initially provide a coarse estimate; over the set of iterations, the algorithm does converge to the accurate estimators.

There are two steps that dominate the process and they are: the time update and the measurement update; in turn, each step has a set of equations that must be solved to calculate the present state. The following is the algorithm:

1. Predict state
  (3) $${\hat{x}}_{k}^{-}=A{\hat{x}}_{k-1}\;+\;Bu_{k}$$
  (4) $$P_{k}^{-}=A.P_{k-1}A^{T}+Q$$
2. Measurement Update—Calculate the Kalman gain (weights)
  (5) $$K_{k}=P_{k}{}^{-}H^{T}{\left[HP_{k}{}^{-}H^{T}+R\right]}^{-1}$$
  $K_{k}$: Kalman gain—The main and unknown value in this equation
3. Update state
  (6) $${\hat{x}}_{k}={\hat{x}}_{k}^{-}+K_{k}\left(z_{k}-H{\hat{x}}_{k}^{-}\right)$$
4. Update state covariance
  (7) $$P_{k}=\left[I-K_{k}H\right]P_{k}{}^{-}$$
5. Loop (now *k* becomes k+1), which is the next and subsequent iterations.
  where:
  $P_{k}{}^{-}$: Prior error covariance Matrix,
  P : Current Covariance Matrix, updated during each iteration,
  Q : Covariance Matrix,
  R : Measurement Noise Covariance Matrix.

This filter’s output is the result of the state update and state-covariance update equations. These provide the combined estimate from the prediction model and measurements from sensors. The mean value of the distribution for each state variable is provided by state matrix and the variances by the covariance matrix. A set of measurements are taken in the present state. The system initializes many matrices. The state variables $x_{0,0}$ can be set based on the initial measurements from the sensors. The covariance of the state can be initialized using the identity matrix I or the covariance matrix Q. Initially, the covariance matrix is not stable but will stabilize as time progresses and the system runs. Measurement noise covariance R matrix is calculated using calibrations performed earlier. The measurement sensors will be developed to measure a large number of readings of the *ground truth state*, from which the variances can be calculated. The variance of the measurements provides the value of $\sigma_{n}^{2}$ in R.

Using literal interpretation(s) from state transition, equations can be used to place the much-needed bounds on dynamic noise. This is because it will be harder to calculate the dynamic noise covariance Q. For instance, 3 sigma in $\sigma_{a}^{2}$ in Q can be calculated by interpreting the target acceleration as a constant velocity model with dynamic noise.

The relative ratio of the measurement noise to the dynamic noise is an important factor. This helps calculate the gains. In the Kalman Filter, it is known to keep one of the noise covariance matrices constant while adjusting the other continuously until the desired performance is achieved. The family of Kalman Filters is to be used in systems that can be run continuously for better accuracy or performance and cannot be used for quick/few iterations since it takes several iterations just to stabilize while using Kalman Filters.

The Kalman filter can become very inefficient and the convergence to the required values can take several steps; to reduce this, i.e., for the system to convergence in fewer steps, the system must be modeled more elegantly and precise estimation of the noise must be achieved.

#### 3.5.2. Extended Kalman Filter

The world functions mostly in a nonlinear manner. Hence, if the techniques used to measure, estimate, predict, analyze, etc. are nonlinear, it is practical, convenient, or accurate. This applies to Kalman Filter as well. The nonlinear filtering problem heuristic is the Extended Kalman Filter (EKF). This technique is naturally the most sought after filtering and estimation for nonlinear systems.

The EKF is based on linearizing dynamics and output functions at an existing estimate(s). In an EKF, the state distribution is usually approximated by a Gaussian Random Variable (GRV), which is then analytically propagated through a first-order linearization of the given nonlinear system under consideration [141,142,143,144]. For example, it functions by propagating an approximation of the conditional expectation and covariance [141,142,144,145,146,147].

#### 3.5.3. Unscented Kalman Filters

Unscented Kalman Filters (UKF) belong to the class of filters called Linear Regression Kalman Filters. These filters are also called Sigma-Point Kalman Filters [148,149]. This type of filter linearizes a nonlinear function of a random variable using a linear regression algorithm between **n** points drawn from the previous distribution of the given random variable. This is also called statistical linearization.

We have seen that the EKF propagates the state distribution through the first order linearization; this may corrupt the posterior mean and covariance. The flaws of EKF have been highlighted by Wan et al. [150]. The UKF is robust to this issue since its derivative-free and uses a deterministic sampling [151]. This logic chooses a set of points called sigma points to represent the state distribution. UKF has an additional step in the selection of sigma points. Broadly, the following are the steps involved:

- Select sigma points
- Model forecasting
- Data assimilation

When data in the input system is symmetric, a deterministic sampling of the data points can approximate the probability density in which the underlying distribution is Gaussian. The nonlinear transformation of the points is an estimation of the posterior distribution. Julier and Uhlmann [148,149,151] state that Unscented transformation is

*Founded on the intuition that it is easier to approximate a probability distribution than it is to approximate an arbitrary nonlinear function or transformation*

#### 3.5.4. Distributed Kalman Filter

Over the past decade, a new technique of filtering that can be used in distributed and dynamic systems has been proposed by Olfati-Saber [91,152]. Techniques of consensus are used to fuse and filter the sensor data and apply covariance information to sensor networks with varying observation matrices. They prove that this provides a collective observer for the processes in the environment that the model uses. They propose a continuous-time distributed Kalman Filter (DKF) that performs a local mean of the sensor data but reaches a consensus with other agents/nodes in the selected network. The above authors also proposed a micro Kalman filter technique wherein an embedded low pass and bandpass consensus filter was used. The consensus filters performed a fusion of the sensor data and co-variance data measured at each agent/node.

Broadly, there are two types of the DKF from the above author:

- Consensus on Estimates
  1. Local Kalman filtering
  2. Continuous-time Distributed Kalman filtering
  3. Iterative Kalman-Consensus filtering
- Consensus on sensor data fusion

Carli et al. proposed a distributed Kalman Filter based on consensus strategies [99], wherein they estimate the state of a dynamic system from distributed noisy measurements. Every agent/node constructs a local estimate based on its individual measurements and also estimates from its neighbors (connected agents). They perform this over a two-step process: the first one being a Kalman based measurement update and the second one being an estimate fusion that uses a consensus matrix. They document that optimizing the consensus matrix for fast convergence.

Spanos et al. proposed a DKF techniques in their research [153] in 2005. The performance of an approximate DKF is analyzed in this research. This technique admits systematic analysis of quantities of several networks like connection density, bandwidth, and topology. The contribution is a frequency domain characterization of the steady-state performance of the applicable DKF. They demonstrate a simple error transfer function with a bound while incorporating the connection density, network topology, and communication bandwidth that performs better using their approach.

Mahmoud et al. performed a review of the DKF during 2013 [100], wherein they compared a centralized Kalman Filter with a distributed Kalman Filter and bring out DKF’s advantages, its techniques, challenges involved, and applications.

Julier et al. wrote a handbook highlighting decentralized data fusion (DDF) with co-variance intersection. This follows a distributed framework in the area of control and estimation. The DDF provides increased robustness and scalability as compared to centralized versions. They state that the time required to implement new computational and sensing components is reduced using DDF.

Recent studies have been performed including optimization of several factors. Some include DKF with finite-time max consensus, DKF over networks with random link failures, etc. These studies suggest that the techniques of DKF are vital in the field of autonomous systems to optimize the system, reduce noise and optimal estimation, etc.

#### 3.5.5. Particle Filters

Particle filters were first introduced in 1993 [154], and have continuously become a very popular class of numerical methods for optimizing the solution of nonlinear non-Gaussian scenarios [31,155,156]. While Kalman filters are linear quadratic estimators(LQE), particle filters, like any member of the family of Bayes filters such as Kalman filters and Hidden Markov Model(HMMs), estimate the posterior distribution of the state of the dynamical system conditioned on the data:

(8) $$\pi \left(x_{1:n}\right)=\frac{\gamma_{n}\left(x_{1:n}\right)}{Z_{n}}$$

where $\pi (x_{1:n})$ is a sequence of target probability densities with increasing dimension, in which every distribution $\pi (x_{1:n})$ is defined through the space $\chi^{n}$.

We need to know only: $\gamma_{n}:\chi^{n}⟶R^{+}$. $Z_{n}$, which is a normalizing constant is given by:

(9) $$Z_{n}=\int \gamma_{n}\left(x_{1:n}\right)\;dx_{1:n}$$

Note that $Z_{n}$ may be unknown. The particle filter provides an approximation of $\pi_{1}\left(x_{1}\right)$ and an estimate of $Z_{1}$ at time 1. Then, an approximation of $\pi_{2}\left(x_{1:2}\right)$ is also an estimate of $Z_{2}$ at time 2.

Considering the simplest implementation wherein $\gamma_{n}\left(x_{1:n}\right)=p\left(x_{1:n},y_{1:n}\right)$, we find that it yields $\pi_{n}\left(x_{1:n}\right)=p\left(x_{1:n}|y_{1:n}\right)$ and $Z_{n}=p\left(y_{1:n}\right)$

Broadly, there are three steps involved in implementing a particle filter [157,158]. They are:

1. Importance sampling:
  - Sample the present trajectories and update
  - Normalize the weights
2. Selection:
  - Samples that have high importance weights are multiplied
  - Samples that have low importance weights are suppressed
3. Markov Chain Monte Carlo Transition:
  - Apply Markov transition kernel with an invariant distribution that is given by $p({x_{0:t}}^{\left(i\right)}|y_{1:t})$ and obtain $\left(x_{0:t}^{\left(i\right)}\right)$

In comparison with standard approximation methods, such as the popular Extended Kalman Filter, the principal advantage of particle methods is that they do not rely on any local linearization techniques or any crude functional approximation [158,159]. They can be used in areas like large systems, where Kalman Filters tend to fail [160]. This technique, however, has its drawbacks, which are expensive computational processes and complexity. Back in 1993, this was an issue, but, nowadays, we can make use of CPU, GPU, and similar high power computing to reduce the computational effort. One of the main deficiencies in a particle filter is that: *Particle filters are insensitive to costs that might arise from the approximate nature of the particle representation*. The other is that, in uninformative sensor readings, samples tend to congregate and a process that times how long it takes for the samples to congregate is essential.

### 3.6. *Research Patents in Data Fusion*

Some of the patents in this research area of data fusion have been as follows:

- **Publication number: US9128185B2**, Publication date: 08 September 2015, Inventors: Shuqing Zeng at GM Global Technology Operations LLC, Title: Methods and apparatus of fusing radar/camera object data and LiDAR scan points
- **Publication number: US20100157280A1**, Publication date: 24 June 2010; Inventors: Kresimir Kusevic, Paul Mrstik, Len Glennie
- **Publication number: WO 2016/100814 A1**, Publication date: 23 June 2016, Inventors: Michael J. GIERING, Kishore Reddy, Vivek Venugopalan, Title: Multi-Modal sensor data fusion for perception systems
- **Publication number: EP 3396408 A1 20181031 (EN)**, Publication date: January 2013, Title: LiDAR and camera data fusion for automated vehicle

## 4. Autonomous Navigation

Robot navigation has been extensively studied in the community for several decades [161,162,163,164,165,166,167]. It can be termed as the safe mobility of the robot from a source location to a target location, without hurting people or properties in its environment, and without damaging itself, and these tasks are performed with no or limited need for a human operator. This means that the navigation system is also responsible for decision-making capability when the system faces situations (critical or otherwise) that demand negotiation with humans and/or other robots. Autonomous navigation is a task that takes in the output from a sensor data fusion module. The Kenneth Research Group performed a detailed study about the future of Autonomous Navigation and state [168].

Autonomous navigation means that a vehicle can plan its path and execute its plan without human intervention. An autonomous robot is one that not only can maintain its stability as it moves, but also can plan its movements. They use navigation aids when possible, but can also rely on visual, auditory, and olfactory cues. The Global Autonomous Navigation Market was valued at USD $2.52 Billion in 2019, and it is further estimated to grow at a CAGR of 16.2% from 2019 to reach USD $6.15 Billion by the year 2025. The Asia Pacific Autonomous Navigation Market is excepted to develop at the most elevated CAGR during the forecasted period 2019–2025.

Research group BIS performed an analysis on the Global Vision and Navigation System Market for Autonomous Vehicle: They focused on Components (Camera, LiDAR, Radar, Ultrasonic Sensor, GPS, and IMU), Level of Autonomy, and Region and quotes [164]:

The automotive industry is on the verge of a revolution with the gradual development of self-driven vehicles. The global vision and navigation system industry for autonomous vehicle depicts the market that is expected to witness a CAGR of 26.78%, during the forecast period from 2019 to 2024.

Autonomous navigation is a formidable task that entails steering the vehicle, registering obstacles all around the vehicle, focusing on the speed at which the vehicle travels, ensuring the destination is reached before the fuel is exhausted, and so on. Other autonomous mobile systems usually have similar tasks but of varying magnitudes. This review focuses on using sensing technology for the three main tasks that are typically part of autonomous navigation. These tasks are *Mapping, Localization, and Obstacle avoidance*. We will review these tasks in greater detail. The three tasks can also be interpreted as the following process(es).

The availability of new-age sensors, advanced computing hardware, and algorithms for processing and fusion of data have made an extremely complex task of information fusion relatively easier to accomplish. This is because, in the past, due to limited computing capabilities, lower sensing quality of then available sensors or exorbitant cost of adequate computing or high-quality sensors, researchers like Brooks [169] chose to develop and use technologies like subsumption architecture that could be implemented on small computers without the use of its memory or storage. Decision-making relies on data fusion which comprises combining inputs from various sources to get a more accurate combined sensor data as output [35,38,44,51]. Each sub-system is detailed below.

### 4.1. Mapping

The task of mapping senses the environment that the robot operates in and provides data to analyze it for optimal functioning. It is also a process of establishing a spatial relationship among stationary objects in an environment. Efficient mapping is a crucial process that gives rise to accurate localization and driving decision making. Usage of LiDARs for mapping is beneficial as they are well known for their high-speed and long-range sensing and hence long-range mapping, while cameras RGB, and RGB-Depth are used for short-range mapping and also used to efficiently detect obstacles [170], pedestrians [171,172], etc. There are various mapping techniques of which topological, metric, and hybrid are more useful than others and hence highlighted in this survey.

#### 4.1.1. Topological Mapping

Topological mapping is usually represented as graphs and is based on connectivity, the environmental structure, and dense surface information [173]. The positional information in these maps do not correlate to the real world; they are mere representations of their existence. Topological approaches [173,174,175] represent robot environments as graphs. The nodes represent situations, areas, or objects (landmarks) (such as doorways, windows, and signboards). The nodes are interconnected by arcs if the two nodes have a direct path between them. Both these robot mappings have demonstrated orthogonal strengths and weaknesses. Occupancy grids are easy to construct and maintain in large-scale environments [176,177] and establish different areas based on the robot’s geometric position within a global coordinate frame. The position of the robot is incrementally estimated using the odometric information and sensor readings taken by itself. Thus, the number of sensors readings that are unbounded are utilized here to determine the robot’s location. Topological approaches determine the position of the robot relative to the model primarily based on the environment’s landmarks or distinct, the temporal sensor features [176]. For example, if the robot traverses two places that seem identical, topological approaches often have difficulty determining if these places are the same or not especially if they have been approached through different paths. In addition, since sensory input usually depends strongly on the robot’s viewpoint, if its sensory input is ambiguous, topological approaches may fail to recognize geometrically nearby places even in static environments, making it difficult to construct large-scale maps. This limitation is reduced in topological approached by their compactness. The resolution of topological maps corresponds directly to the complexity of the environment. The compactness of topological representations gives them three key advantages over other approaches: (i) fast planning, (ii) interfacing to symbolic planners and problem-solvers, and (iii) natural interfaces for a human speech like instructions (such as “go-to kitchen"). They recover early from slippage and drift since they do not require the exact determination of the geometric position of the robot which must be constantly be monitored and compensated as in a grid-based approach.

#### 4.1.2. Grid Based Approach

Grid-based approaches [178,179,180] represent the robot environments as evenly-spaced grids. Each grid cell may contain a representation of an obstacle or a free path to the target as applicable. Grid-based approaches are hampered by their enormous space and time complexity. This is because the resolution of a grid must be fine enough to capture the details of the robot world [181]. Jiang et al. developed a method to capture the grid maps and then stitch them to generate a larger map [182].

#### 4.1.3. Metric Mapping

Geometric maps are based on the distance, and these map distances correlate and correspond to the distances found in the real world. They can be feature or landmark-based. While landmark needs feature identification or designing the environment, the dense technique is based entirely on the sensors to create the map. These sensors create a geometric representation of the environment surfaces [183,184,185,186]. Other types of mapping are sensor level maps, which are sensor data derivations, and semantic maps, which are high-level decision enabling maps and contain object and space property details.

#### 4.1.4. Hybrid Mapping

Hybrid mapping utilizes a mixed set of properties of any of the above mapping techniques, mainly metric and topological mapping [187]. This technique takes in the best properties, depending on the task, the environment where it is implemented, and develops a map that could be used to accomplish the task.

New techniques in the area of mapping and localization have been developed over the last few decades. Many of these techniques incrementally and iteratively build maps and localize the robot, for every new sensor data scan that the robot accepts [183,185]. The drawbacks of these techniques are their failure when large cyclical scan (open-loop) environments are involved, despite their high-speed processing. Cyclical environments will output cumulative errors that can grow exponentially and without any bounds. This is because, in these environments, backward temporal corrections tend to be time-consuming, and several systems may not be able to achieve acceptable results.

**Figure 6:**
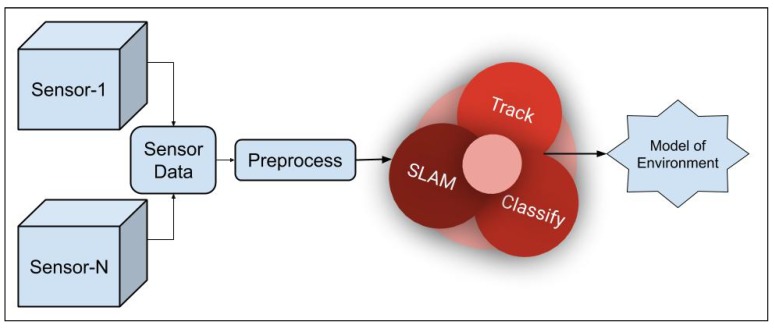
High-level perception task.

Mapping for autonomous mobile vehicles is a discipline related to computer vision [188,189] and cartography [190]. In such environments, one of the preliminary tasks could be the development of a model of the world, using the map of the environment, making use of onboard sensors. The other task would be utilizing the constructed pre-existing map. The map can be developed using SLAM [188,191]. This usage of the a priori information can be called the development of an autonomous vehicle for the known environment. An implementation of slam that utilizes multiple sensors, and their fused data are given in Figure 6.

Constructing a map can be exploratory [192], without the use of any pre-existing mapping information or an existing floor plan that details the presence of walls, floor, walls, ceiling, etc. Using the techniques of exploratory navigation [192], the autonomous vehicle can develop the map and continue to navigate. If the floor plan is available, the system can create the map by traversing along with the building floor map and localize itself. In order to map the environment, a LiDAR can be used which provides a three-dimensional pointcloud of the environment where the robot is situated. Hence, we can define a robotic mapping as that branch of robotics that deals with the study and application of the ability of the robot to construct the map or floor plan, of the environment where it is situated, using its sensors. An area of mapping that deals with the active mapping of the robot in its environment while simultaneously localizing itself is called Simultaneous Localization and Mapping (SLAM) [191,193,194,195,196]. There are various flavors of SLAM like EKF SLAM, FastSLAM (1 and 2), DP-SLAM, Parallel Tracking and Mapping(PTAM), ORB-SLAM, MonoSLAM, and so on. However, a detailed study of SLAM is out of the scope of this survey. Aguilar developed a path planner based on RRT* [197] for real-time navigation.

### 4.2. Localization

Localization is one of the most fundamental competencies required by an autonomous system, as the knowledge of the vehicle’s location is an essential precursor to take any decisions about future actions, whether planned or unplanned. In a typical localization situation, a map of the environment or world is available and the robot is equipped with sensors that sense and observe the environment as well as monitor the robot’s motion [188,198,199,200]. Hence, localization is that branch in autonomous system navigation, which deals with the study and application of the ability of a robot to localize itself in a map or plan.

The localization module informs the robot of its current position at any given time. A process of establishing the spatial relationship between the intelligent system and the stationary objects Localization is achieved using devices like Global Positioning Systems(GPS), odometric sensors, Inertial Measurement Units (IMU), etc. These sensors give the position information of the autonomous system, which can be used by the system to see where it is in the environment or the robot world [198,201,202]. Some important techniques of localization are listed below.

#### 4.2.1. Dead Reckoning

Dead reckoning uses odometric data, trigonometric, and robotic kinematic algorithms to determine the distance traveled by the robot from its initial position. However, two major issues impact their performance. The robot has to know the initial position and the second is the time measurement related errors, which impact the accuracy and sometimes go below acceptable levels. Thrun et al. [203] used a probabilistic method to reduce the errors, known as particle filtering. Others used Extended Kalman Filter [204] and similar techniques to reduce the errors. Researchers utilized sensors like IMU to perform dead-reckoning [205,206], while others used ultrasonic sensors with Kalman filters to improve the measurements [183].

#### 4.2.2. Signal-Based Localization

Sensors that communicate via signals are several [207], of which Radio Frequency Identification (RFID) [208,209], WiFi [210], and Bluetooth [211] are a few. In this technique, the positions of a network of nodes are identified based on distance estimates between them.

#### 4.2.3. Global Positioning

Outdoor navigation is involved in cases of outdoor search and rescue missions. Localization in such cases involves usage of Global Positioning Systems (GPS) that efficiently work only outdoors. GPS technology was first developed by NAVSTAR [212] and is one of the favorite technologies to date for outdoor navigation. Some of the GPS companies are Navstar™, Garmin™, TomTom™, Mobius™, etc. to name a few. GPS provides very accurately (normal range up to to one meter), some advanced GPS provide accuracy up to two centimeters like the Mobius agriculture mapping system [213], which is used on autonomous tractors.

#### 4.2.4. Network of Sensors Localization

A sensor network is comprised of several sensors that can communicate either wirelessly or wired. Choi et al. combined RFID tags with an external camera to monitor the robot [214]. In some cases, ceiling-mounted cameras were used to improve localization when odometry data were fused with LiDAR [215]. The camera was used to locate obstacles and also to aid in the initial position estimation.

#### 4.2.5. Vision-Based Localization

Sensors mounted on the robot provide the latest and accurate data concerning the robot. This system of sensors can be generalized to different environments and robots that use them and hence are sought after in the present research areas. The outdoor environment can be supported by a single or multiple sets of GPS and are fairly accurate. Indoor environments use LiDAR sensors [216] and/or vision-based sensors [217,218].

#### 4.2.6. Indoor VR Localization

Indoor localization uses the new age technologies like Virtual Reality head-sets, and 3D laser sensors are on the rise. One such example is the HTC ViVe™ [219] Lighthouse technology. This system floods a room with light invisible to the naked eye. Lighthouse functions as a reference point for any positional tracking device (like a VR headset or a game controller) to figure out where it is in real 3D space. The lighthouse system shoots light into the world to assist receiving systems localize themselves. The receivers, which are tiny photo sensors that detect the flashes and the laser light, are placed on various locations on the vehicle—in this case, the wheelchair. When a flash initiates, the receiver starts counting until it detects the photosensor situated on it gets hit by a laser beam and uses the relationship between where that photosensor exists on the wheelchair, and when the beam hits the photosensor, to mathematically calculate its exact position relative to the base stations in the room. When we have detection by enough of the photosensors with a laser at the same time, they form a pose that provides the position and the direction of the wheelchair. This is called an *inside-out tracking system* since the headset uses external signals to figure out where it is.

### 4.3. Path Planning

Path Planning is an important subtask of autonomous navigation and is generally termed as a problem of searching for a path which an autonomous system has to follow in a described environment and requires the vehicle to go in the direction closest to the goal, and, generally, the map of the area is already known [220,221,222,223]. Path planning when used in conjunction with techniques of obstacle avoidance [223] gives a more robust deployment of the path planner module by enabling the system to avoid hazardous collision objects, no-go zones, and negative objects like potholes and similar objects.

Path planners can be designed based on the following properties:

1. *Complete or Heuristic*: A complete type of path planner was designed by Wagner et al. [224] in which a multi system path planner uses both coupled and de-coupled algorithms and hence benefits from both of the techniques. Urdiales et al. designed a complete path planner [225] by using a pyramid structure for pre-processing the information to existing classical path planners. Heuristic approaches were applied by Mac et al. [226]. Vokhmintsev [227] designed yet another heuristic path planned that could be used in unknown dynamic environments,
2. *Global or Local*: Global path planners use environment information available apriori to navigate. Information about the environment will be known a priori and can consist of maps, cells, grid, and so on. A complete path is generated from source to target, before the vehicle starts moving [228]. Some of the global planners are Voronoi [229] by Bhattacharya et al., Silhouette [230] by Canny et al., Dijkstra [231] by Skiena et al., A${}^{*}$ by Dechter et al. [232], Neural Network based by Yang et al. [233], and so on.
  A local path planner was proposed by Buniyamin et al. [220] in which they use bug algorithm to detect obstacles in the environments using onboard sensors and plan the path. This is a local planner that uses obstacle border to guide the vehicle towards the target, until the required target achievement conditions are met. They propose a new algorithm ’PointBug’ that minimizes the use of the border (outer periphery), in order to generate a path from source to target. Some of the local path planners are based on [228] Splines as given by Piazzi et al. [234], Bezier lines as given by Rastelli et al. [235], arcs and segments by Reeds et al. [236], Clothoids lines [237], and so on.
3. *Static or Dynamic*: When an autonomous system encounters static objects in its path, it can perform static path planning and, if it encounters moving objects, it performs dynamic path planning.
  Kumar et al. did initial research on static and dynamic path planners on humanoid robots [238]. They developed a novel controller that represents static path planner as a single robot encountering random static obstacles and dynamic planner as multiple robots encountering random static obstacles. They use a Petri-net controller. Tuba et al. [239] developed an optimal path planner that encounters static obstacles. They used harmony search algorithm and adapted it to their requirements for static obstacles and danger or no-go zones. Dutta et al. [240] developed a static path planner for snake-like robots when they encounter static obstacles using a critical snakeBug algorithm.
  As recent as 2020, Gabardos et al. [241] discussed the methods for a variant of dynamic path planning that were based on multisensor fusion to detect the pose, size, and shape of the object along the planned route. The dynamic routing is accomplished by interpolation of the route poses, with some being re-positioned. Connell et al. developed dynamic path planners [242] for mobile robots with replanning using RRT. Liu et al. [243] developed a dynamic path planner using an improvized ant colony optimization algorithm. They simulate the algorithm on a grid map.

### 4.4. Obstacle Avoidance

For successful navigation of an autonomous system, avoiding obstacles while in motion is an absolute requirement [30,32,33,35,170]. The vehicles must be able to navigate in their environment safely. Obstacle avoidance involves choosing the best direction among multiple non-obstructed directions, in real-time, hence obstacle avoidance can be considered to be more challenging than path planning.

Obstacles can be of two types (i) Immobile Obstacles (ii) Mobile Obstacles. Static object detection deals with localizing objects that are immobile in an environment—for example, of indoor static obstacles, can be a table, sofa, bed, planter, TV stand, walls, etc. Outdoor static obstacles can be buildings, trees, parked vehicles, poles (light, communication), (standing or sitting) persons, animals lying down, etc. Moving object detection deals with localizing the dynamic objects through different data frames obtained by the sensors to estimate their future state example of indoor moving objects can be walking or running pets at home, moving persons, operating vacuum robots, crawling baby, people moving in wheelchairs, etc. Outdoor moving obstacles can, for instance, be moving vehicles, pedestrians walking on the pathway, moving ball thrown in the air, flying drone(s), running pets, etc. The object’s state has to be updated at each time instance. Moving object localization is not a simple task even with precise localization information. The challenge increases when the environment is cluttered with obstacles. The obstacles can be detected using two approaches that rely on prior mapped knowledge of the targets or the environments [33,37,48,180,244]. These are the (i) Feature-based approaches that use LiDAR and detect the dynamic features of the objects; and (ii) Appearance-based approaches that use cameras and detect moving objects or temporally static objects.

The task of obstacle avoidance keeps the vehicle from colliding with obstacles and keeping the vehicle in a safe zone. It is a process that starts with identifying objects that are present in the environment and obstacle avoidance is a critical component of autonomous system navigation [170]. Autonomous vehicles must be able to navigate their environment safely. We can broadly classify obstacle avoidance into static and mobile obstacle avoidance [245,246]. As the name suggests, static obstacle avoidance deals with navigating around obstacles that do not move and only the autonomous vehicle are in motion. Static obstacle avoidance is a process of establishing the temporal and spatial relationship between the mobile vehicle and the immobile obstacles—for example, a sofa in a living room. In contrast, mobile obstacle avoidance is a process of establishing the temporal and spatial relationship between the mobile objects in the environment, in addition to the vehicle and stationary objects. While path planning requires the vehicle to go in the direction nearest to the goal [223], and generally the map of the area is known, obstacle avoidance entails selection of the best direction among several *unobstructed directions* in real time.

Any autonomous system, or autonomous navigation function based system, must be aware of the presence of obstacles. When such a system deals with human assistance, the obstacle problem becomes even more critical, since there is zero-tolerance for failure. Objects are detected, identified and deemed as obstacles by the system. The obstacles can either be static or mobile. If it is a static obstacle, the problem reduces to the detection of present position and avoidance. If the obstacle is mobile, an autonomous system should not only know where the obstacle currently is but also track where the obstacle could be in the near future. This reason prompts us to perceive the obstacles as dynamic entities and the task of obstacle avoidance is a complex one.

There are several existing approaches for solving the obstacle avoidance problem; some commonly used approaches are the traditional object detection through Vector Field Histogram (VFH) [34,180,247], the Dynamic-Window Approach [248] and occupancy grid algorithm [170,249], and the Potential field method [250]. The classification and localization of every object of importance and interest are necessary for the obstacle detection and avoidance tasks for a robot that uses cameras. Some of the traditional methods use Histograms [34,180,247] and have provided good results. However, techniques using Neural Network (NN) or Deep Learning(DL) have continually been outperforming them like passive DL techniques given in [251,252] to name a few. There are real-time NN techniques like [253] that can detect much quicker compared to the traditional techniques. Recent research has produced two fundamental paradigms for modeling indoor robot environments: *the grid-based paradigm and the topological paradigm*.

Grid-based approaches [178,179,180] represent the robot environments as evenly-spaced grids. Each grid cell may contain a representation of an obstacle or a free path to the target as applicable. Topological approaches [173,174,175] represent robot environments as graphs. The nodes represent situations, areas, or objects (landmarks) (such as doorways, windows, signboards). The nodes are interconnected by arcs if the two nodes have a direct path between them. Both these robot mappings have demonstrated orthogonal strengths and weaknesses. Occupancy grids are easy to construct and maintain in large-scale environments [176,177] and establish different areas based on the robot’s geometric position within a global coordinate frame. The position of the robot is incrementally estimated using the odometric information and sensor readings taken by itself. Thus, the number of sensors readings are unbounded and are utilized here to determine the robot’s location.

Contrary to this, topological approaches determine the position of the robot relative to the model primarily based on the environment’s landmarks or distinct, the temporal sensor features [176]. For example, if the robot traverses two places that seem identical, topological approaches often have difficulty determining if these places are the same or not especially if they have been approached through different paths. In addition, since sensory input usually depends strongly on the robot’s viewpoint, if its sensory input is ambiguous, topological approaches may fail to recognize geometrically nearby places even in static environments, making it difficult to construct large-scale maps. Contrary to this, grid-based approaches are hampered by their enormous space and time complexity. This is because the resolution of a grid must be fine enough to capture the details of the robot world. This limitation is reduced in topological approached by their compactness. The resolution of topological maps corresponds directly to the complexity of the environment. The compactness of topological representations gives them three key advantages over grid-based approaches: (i) fast planning, (ii) interfacing to symbolic planners and problem-solvers, and (iii) natural interfaces for a human speech like instructions (such as “go-to kitchen”). Topological maps recover early from slippage and drift since they do not require the exact determination of the geometric position of the robot which must be constantly be monitored and compensated as in a grid-based approach.

## 5. Fusion of Sensor Data for Autonomous Navigation

This section discusses how to use output of fusion in autonomous navigation and its related sub-tasks as highlighted in Section 4.

### 5.1. Mapping

Thrun et al. (2000–2002), presented a novel algorithm which is strictly incremental in its approach [189,203]. The basic idea is to combine posterior estimation with incremental map construction using maximum likelihood estimators [165,176]. This resulted in an algorithm that can build large maps in cyclical environments in real-time, even on a low footprint computer like a micro-computer e.g., Odroid XU4. The posterior estimation approach enables robots to localize themselves globally in maps developed by other linked robots and thus making it possible to fuse data collected by more than one robot at a time. They extended their work to generate 3D maps, where multi-resolution algorithms are utilized to generate low complexity 3D models of indoor environments:

(10) $$m_{t}=\left\{\langle O_{\tau },{\hat{s}}_{\tau }\rangle \right\}\;\;where\;\;\tau =0,1,2,3..t$$

where: $O_{\tau }$: laser scan

${\hat{s}}_{\tau }$: laser scan’s pose

τ: time index

(11) $$\underset{x}{\arg\;\max}\;P\left(m|d_{t}\right)$$

where data $d_{t}$ are a sequence of LiDAR measurements and odometry readings $d_{t}=\left\{s_{0},a_{0},s_{1},a_{1},..s_{t},a_{t}\right\}$,

where $s_{\tau }$ denotes an observation (laser range scan), $a_{\tau }$ denotes an odometry reading, and *t* and τ are time indexes. It is assumed that observations and odometry readings alternate each other.

The assumption is that, when a robot receives a sensor scan, it is not likely that an obstacle is perceived in future measurements when it scans space previously perceived as free. The likelihood is inversely proportional to the distance between previous and current measurements:

(12) $${\hat{s}}_{t}=\underset{s_{t}}{\arg\;\max}\;P\left(s_{t}|o_{t},a_{t-1},{\hat{s}}_{t-1}\right)$$

The results are determined using a gradient ascent algorithm. The result of the search, ${\hat{s}}_{t}$, and its corresponding scan $o_{t}$ are appended to the map.

As recent as 2019, Akhtar et al. [254] developed a data fusion system that was used to create a 3D Model with a depth map and object 3D reconstruction. Jin et al. [255] proposed an approach for SLAM using 2D LiDAR and stereo camera with loop closures to estimate odometry. As recent as 2020, Andersen et al. have used LiDAR and camera fusion for fast and accurate mapping in autonomous racing [256]. They develop a planning pipeline in addition to perception and mapping and implement it on an autonomous race car, for the "Formula Student Germany(FSG) driverless competition" and placed first.

### 5.2. Localization

Localization of an autonomous vehicle typically uses sensors like GPS, odometric, IMU with magnetometer, accelerometer, and so on. The data fusion in these sensors is challenging due to the presence of drift, as in a GPS module. The data fusion should also consider the drift and counter it with applicable measurements in order to have the system localize itself accurately. Section 3.3 provides one of the available techniques for the implementation of an input–output method of data fusion, first proposed by Dasarathy et al. [96]. After the data are successfully fused in the perception module, the information is passed on to the control module and the control module uses this information in an iterative manner. When the data fusion system detects an obstacle, it passes this information as well to the controller, and it invokes the obstacle avoidance segment as required.

As a second example, consider simultaneous localization and mapping (SLAM). In SLAM, the integrated output of the perception module is input to Zhang et al. [257], who proposed a robust model that used the MM-estimate technique for segment-based SLAM in dynamic environments. The raw 2D laser rangefinder data were split into laser segments and enhanced with outliers of the moving objects. However, they state that the SLAM performance would deteriorate if the moving objects start and stop often for short intervals, as they may be misrepresented as features. This is because the monocular camera lines are mostly static after the required processing. They mitigate this by integrating the laser segments with line features and removing the pseudo segments using Bayesian techniques.

They improved this technique using MPEF-SLAM [258] wherein they implemented the state estimates from each of the monocular cameras and the LiDAR SLAM. This increased the accuracy of localization as it reduced the covariance of the robot pose.

**Figure 7:**
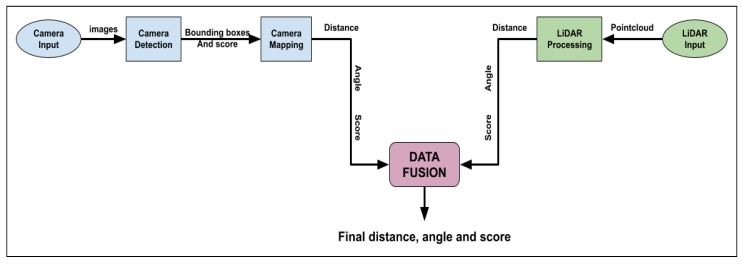
High level block diagram of LiDAR and Camera data fusion [259].

As part of detection research, Wei. et al. [259] fused LiDAR data and camera data using fuzzy logic and progressed to successfully implement SLAM and eventually perform detection of obstacles. A high-level block diagram is given in Figure 7, the information is passed on to the control module, and the control module iteratively uses this information. When the data fusion system detects an obstacle, it passes this information as well to the controller, and it invokes the obstacle avoidance segment as required.

### 5.3. Path Planning

As mentioned in the previous section(s), path planning is an important task in autonomous navigation in which a system can perform global planning using pre-existing maps or local planning when no maps exist a priori. This means that the path planning is dependent on mapping. In cases where the autonomous vehicle encounters static or moving obstacles, it uses obstacle avoidance techniques. Hence, the usage of sensors is vital.

Wang et al. [260] developed a vision based sensor fusion platform for path planning on a mobile robot. They use a pseudo-range processing method for vision based sensor fusion using heterogeneous sensors. They also use precise GPS, inertial and orientation sensors.

Ali et al. [261] developed an approach for a three-wheeled mobile robot in an online navigation of road following and roundabout environments. They developed a complete planner in which the sensor fusion was used to remove noise and uncertainties from the sensors. The motion controller was used to control the kinematics of the vehicle by using a resolved acceleration control integrated with an active force controller to reject high disturbances. Gwon et al. [262] developed sweeper robots for the curling Olympic games by developing a sensor fusion system that inputs to a path planner based on path estimation of a curling stone. The task of the robot was to clear the path efficiently so that the curling stone reaches its intended location. The trajectory of the stone was calculated/recalculated in an optimal time step using the trend-adjusted exponential smoothing method. We see that path planning and obstacle avoidance was key and they relied on the on-board sensors to provide the optimum situation awareness to achieve the task.

Xi et al. [263] proposed a mapping approach to improve the accuracy of the robot swarm navigation by using a grid-map that used multi-sensor data fusion. They also proposed a path planning algorithm based on an improved intelligent water droplet algorithm. Their data fusion framework comprises of radar and depth camera sensors. They system verified the map construction based on the fused sensor data.

Sabe et al. [264] used occupancy grids to find the path from robot source or current location to its goal; using this, the robot can safely reach the target location. They achieve this by defining every occupancy grid cell as a node that connects to a neighboring cell and also define the path planning problem as a search problem, using an A* search algorithm.

### 5.4. Obstacle Avoidance

In addition to cameras, LiDARs can be used to detect objects. A 3D point cloud is an output from the LiDAR. For efficient operation, the autonomous vehicle needs accurate data from each of its sensors. The reliability of the operation of an autonomous vehicle is hence proportional to the accuracy and hence the quality of the associated sensors. Each type of sensor has its own limitations. Table 2 gives a comparison of the sensor types and their properties that are useful for navigation tasks. Given below are some of their specific limitations:

- LiDAR: Weather phenomena as in rain, snow, fog [265]
- Stereo vision: Distance from target, Baseline [266]
- Ultrasound: Pollutants [267]

Sensor data fusion is effective whenever multiple sensors (homogeneous or heterogeneous) are utilized and data fusion is not limited to the field of robotics [214] and in fact surveillance [268], gesture recognition [18], smart canes [7], guiding glasses [269] use this concept efficiently. The effective temporal, spatial and geometrical alignment of this suite of heterogeneous sensors and the diversity utilization is called sensor data fusion [38,39]. Depth perception cameras provide limited depth information in addition to data-rich image data. Although cameras have the advantage of providing extremely rich data almost equivalent to the human eye, they need significantly complex machine vision techniques that require high computing power. In addition to his challenge, the operational limitation can be attributed to adequate lighting and visibility. Cameras are used very efficiently in detecting sign recognition, pedestrian detection [171,270], lane departure [271], identification of objects [116,272,273]. Cameras are much cheaper compared to radars or LiDARs [28]. Hence the community prefers them over other sensors in certain applications. Both LiDARs and Depth Cameras contain depth-sensing sensors. While the cameras estimate the depth information using disparity information in the image, the LiDAR generates depth information from the environment. Each sensor has its pros and cons. The depth cameras provide rich depth information, but their field of view is quite narrow. In contrast, the LiDARs contain an excellent field of view but do not provide rich environment information and instead provide sparse information [214,269,274]. The LiDAR provides information in the form of point cloud while the camera gives luminance We can see that these sensors can complement each other and can be used in complex applications. This is the advantage that we focus on in this study. Caltagirone et al. successfully developed a neural network that detected the road [93]. They projected an unstructured and sparse point cloud on the camera plane and un-sample it to obtain a set of dense 2D images. Multiple CNNs were trained to detect the roads. They found out that the fused data from the two sensors were better in terms of data accuracy and detail as compared to the individual sensors.

Huber et al. studied LiDAR and camera integration [275] and found that the sparse information in the LiDAR may not be useful for complex applications and that a data fusion with a sensor that has rich information is useful. They also establish that stereo vision camera performs poorly in areas without texture and scenes containing repetitive structures, and hence its subsequent fusion with LiDAR leads to a degraded estimation of the 3D structure. They proved that fusing the LiDAR data directly into the depth camera reduces false positives and increases the disparity image density in the texture-less surface and hence reducing the disparity space. They devised a method to use the LiDAR information and then deduce the most optimum disparity information per pixel in the image. The advantages this provides are reduced computation and better disparity image quality. An added advantage is path propagation since we can predict the expected or final disparity and the related gradient.

Banerjee et al. developed a data fusion system of online camera and LiDAR data. Instead of using an exhaustive grid search for extrinsic calibration, they used a gradient-free optimizer [276]. This gives their technique a low footprint, a lightweight quality, and the ability to execute in real time on an onboard computer on the vehicle. Recently, Manghat et al. developed a real-time tracking system that used LiDAR and camera in early 2020 [277]. They focus on tracking in this research due to its importance in autonomous navigation assistance systems like active driver assistance systems (ADAS), forward collision warning system (FCW), adaptive cruise control, and collision by breaking (ACCCB). The optimal state of the objects is estimated by obtaining the states of each sensor and then fusing them to improve the state estimations of the objects in the environment. Asvadi et al. developed a multimodal vehicle detection system by fusing RGB camera and 3D LiDAR data [278] in 2018. This was used in identifying obstacles surrounding the autonomous vehicle. Three modalities such as *a dense map (DM) consisting of the LiDAR’s sparse data which was an upsampled output, high-resolution map from the LiDAR’s reflectance data called Reflectance Map (RM), and RGB image from a monocular camera extrinsically calibrated to the LiDAR* the three sources of data were input to the CovNet detectors and later integrated to improve the detection.

After a successful data fusion, the output of the fusion can be used to detect objects. There is a substantial list of detection algorithms and [32,170,251] they can very efficiently detect objects in the environment where the autonomous vehicle operates. As an example, consider an autonomous wheelchair that operates in a known environment, i.e., an environment has been mapped and the vehicle needs to navigate to known destinations. If the environment does not change, the operator of the vehicle may just use the stored navigation routes and reach the destination from the source—for example, the living room to the kitchen. However, in an environment like a house, obstacles like chairs may have been moved, a child could be playing in the living room, or an assistive dog may be lying on the floor and resting. These could be termed as obstacles that the vehicle needs to avoid, or it will end up harming the child, pet, or operator. Hence, the need for the vehicle to operate with accurate situation awareness (SA) information. For efficient SA, the wheelchair may need to deal with a two-tier sensor data fusion. The first tier could be the outer loop of the LiDAR that detects the distant objects, obstacles, etc. The second tier could be, for instance, a stereo vision camera Realsense D435 output [127], which could be used for immediate object detection, recognition, and avoidance as needed. There are many classical methods for the detection of objects in an image, such as dense image pyramids and classifier pyramids [279]. Various feature detection methods such as fast feature pyramids that can quickly calculate places in the image where there could potentially be a person [279]. The Speed is around 30 Frames per second. In addition, we reviewed R-CNN and their variants, including the original R-CNN, Fast R-CNN [280], and Faster R-CNN [253], Single Shot Detector (SSDs) [281], and a Fast version of You Only Look Once (YOLO-Fast) [251,252].

**Figure 8:**
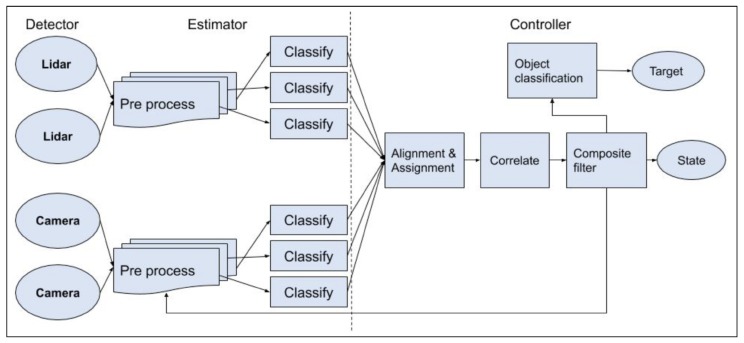
Architecture of a fusion system.

A high-level architecture with post-classification fusion in an autonomous system where the core of the fusion is performed after the classification is given in Figure 8. The raw signal is sensed and processed. Using classification techniques using technologies like YOLO, a preliminary classification can be performed. The KITTI provides benchmark [282] results. Qi et al. [36] performed an object classification for 3D object detection using RGB-D data and Complex-YOLO technique, a flavor of fast YOLO by Simon et al. [283]. This first level of classification is performed on the data and features are extracted. It is fed through an alignment process, in order to correlate the LiDAR data points with the stereo vision camera pixel data. Finally, a second classification is performed using the features, in order to extract the details of the objects.

Dynamic obstacle avoidance techniques like the dynamic window approach to collision avoidance by Fox et al. [248] or the real-time obstacle dependent Gaussian obstacle avoidance system Potential Field [250] use the principles of real-time situation awareness and dynamic obstacle avoidance to provide safe operation in a hazardous environment. Dynamic obstacle avoidance demands a true real-time behavior-based system to sense the environment of the autonomous vehicle.

Table 3 provides a high level summary of the data fusion for the respective sub tasks of autonomous navigation that are mentioned in this section. The aim is to provide a gist of the research and the respective researchers who have used data fusion and the respective sub task of navigation. We strongly recommend referring to Section 2, Section 4, and Section 5 to get a holistic perspective of data fusion for navigation. If the reader wishes to know about the hardware, Section 3 will be useful.

## 6. Conclusions

As part of this survey, we have briefly introduced sensor data fusion and autonomous navigation. We have reviewed the most popular data fusion techniques that can be used in navigation tasks for intelligent mobility systems. This survey is by no means exhaustive, due to the nature of the research area. However, it provides adequate information to the audience by reviewing the laser and optical sensors like LiDAR and camera, respectively. A brief look into the task of autonomous navigation, while explaining its sub-tasks namely mapping, localization, and obstacle avoidance is accomplished. The multi-disciplinary nature of data fusion was researched, and it was found that multiple sensors are better than one when used for autonomous vehicle tasks like robot navigation. The acute need for a robust data fusion process, methodology, and logic are described, and a discussion of the concepts of robot perception is provided, in addition to presenting some of the previous works that have performed seminal research in this area.

We have observed from research publications how data fusion can drive the future of autonomous systems and extend algorithms into areas of commercial autonomous systems, in addition to military systems. Estimation and filtering techniques such as Kalman filters, particle filters, and similar techniques are briefly discussed and also the need for their usage is provided.

A comparison of the different types of data fusion and their pros and cons are provided as well. Some inexpensive but robust sensors like the Intel Realsense D435 and RPLiDAR were researched, and their performance and capabilities are documented and references to top performers (although expensive sensors) sensors like Velodyne and eclipse are given. As a first look into sensor fusion, calibration techniques suggested by some leading manufacturers are provided. Multimodal sensor architectures are discussed in Section 1 and Section 5. A summary of the application of data fusion for the four sub tasks of navigation is given in tabular form in Table 3, in Section 5. In conclusion, we state again that using a good perception system with an appropriate data fusion system is vital for the optimal functioning of an autonomous system and its task of navigation.

References and Note

## Figures and Tables

**Table 1:** Data fusion techniques and their classifications.

Classification Type	Types of Fusion for the Given Classification				
Classification based on Data Relationship	Complementary data relationship	Redundant duplicated relationship	Co-operation relationship		
Classification based on Abstraction Level	Signal level	Pixel level	Characteristics	Symbols	
Dasarathy classification	Data In Data Out	Data In Feature Out	Feature In Feature Out	Feature In Decision Out	Decision In Decision Out
JDL	Source pre-processing	Object refinement	Situation assessment	Impact assessment	Process refinement
Classification based on Architecture	Distributed	Centralized, Decentralized	Hierarchical		

**Table 2:** Characteristics and properties of acoustic and vision based sensors.

Sensor	Data Density	Low Light Operation	Position Information	Velocity Information	Class	Size Information	Color Availability
LiDAR	$10^{5}$	Yes	Yes		Yes	Yes	
Ultrasonic	$10^{2}$	Yes	Yes		Yes	Yes	
Radar	$10^{3}$	Yes	Yes	Yes	Yes		
Thermal Camera	$10^{5}$	Yes			Yes		Yes
Vision Camera	$10^{7}$		Yes			Yes	Yes

**Table 3:** Summary of the usage of data fusion techniques in autonomous navigation.

Summary of the Usage of Data Fusion Techniques in Autonomous Navigation	
**Mapping**	We discuss the usage of data fusion in mapping applications
	Thrun, S. Survey of Robotic Mapping and discuss their research about how combining posterior estimation with incremental map building using maximum likelihood estimators
	Akthar - Developed a data fusion system that was used to create a 3D Model with a depth map and object 3D reconstruction
	Jin - proposed an approach for SLAM using 2D LiDAR and stereo camera
	Andersen et al., used LiDAR and camera fusion for fast and accurate mapping in autonomous racing
**Localization**	We briefly discuss what sensors are used in localization and the challenges using these sensors in data fusion
	Dasarathi et al., proposed techniques for localization and navigation in general
	Zhang et al., proposed a robust model that used the MM-estimate technique for segment-based SLAM in dynamic environments using 2D LiDAR.
	Wei et al., did data fusion with LiDAR and camera using Fuzzy logic techniques.
**Path Planning**	Wang et al., developed a sensor fusion platform using camera for a mobile robot which can be used for path planning.
	Ali et al., developed a fusion algorithm for an online navigation with a complete planner.
	Gwon et al., designed sweeper robots that were used for path estimation for the curling game
	Xi et al., proposed a swam technique with mapping and path planning to improve navigation.
	Sabe et al., used occupancy grids for path planning and finding paths from robot source to target location
**Obstacle Avoidance**	Several references are provided that introduce various research areas with systems like autonomous, assistive, haptic, motor and optical assistive and similar systems
	We discuss several algorithms by Danescu, wu and redmond and their teams that are used in object detection.
	We discuss pros and cons of each sensor for object detection.
	Banerjee et al., developed a fusion system of LiDAR and camera using a gradient free optimizer giving them a low footprint, lightweight and robust system.
	Huber et al., studied the same senors and performed data fusion. They state that the sparse LiDAR information is not useful for complex applications
	Asvadi et al., researched multimodal fusion and used it in vehicle detection areas, identifying obstacles around an autonomous vehicle
	Manghat et al., developed a real-time tracking fusion system for ADAS systems.
	Luo et al. published a manuscript documenting datafusion techniques, approaches applications and future research.
	Dynamic obstacle detection and avoidance is discussed as proposed by Fox et al., and theGaussian obstacle avoidance system by cho,
	We present the flow of a navigation system, with data fusion feeding into an object detection system.
	We summarize the usage of AI and Neural networks in object detection; techniques like YOLO, SSD, CNN, RNN were discussed
	An architecture of data fusion system, that can be used in autonomous navigation is presented.
